# CAMK2D: a novel molecular target for *BAP1*-deficient malignant mesothelioma

**DOI:** 10.1038/s41420-023-01552-5

**Published:** 2023-07-21

**Authors:** Sivasundaram Karnan, Akinobu Ota, Hideki Murakami, Md. Lutfur Rahman, Md Wahiduzzaman, Muhammad Nazmul Hasan, Lam Quang Vu, Ichiro Hanamura, Akihito Inoko, Miho Riku, Hideaki Ito, Yoshifumi Kaneko, Toshinori Hyodo, Hiroyuki Konishi, Shinobu Tsuzuki, Yoshitaka Hosokawa

**Affiliations:** 1 Department of Biochemistry, , Aichi Medical University School of Medicine, Nagakute, Aichi Japan; 2 Department of Nutritional Environment, College of Human Life and Environment, Kinjo Gakuin University, Nagoya, 463-8521 Japan; 3 Department of Pathology, Aichi Medical University School of Medicine, Nagakute, Aichi Japan; 4 Department of Molecular Oncology, H. Lee Moffitt Cancer Center and Research Institute, Tampa, FL US; 5 Eukaryotic Gene Expression and Function (EuGEF) Research Group, Chattogram, 4000 Bangladesh; 6 Division of Hematology, Department of Internal Medicine, Aichi Medical University School of Medicine, Nagakute, Aichi Japan

**Keywords:** Mesothelioma, Phenotypic screening

## Abstract

Malignant mesothelioma (MMe) is a rare but aggressive malignancy. Although the molecular genetics of MMe is known, including *BRCA1-associated protein-1* (*BAP1*) gene alterations, the prognosis of MMe patients remains poor. Here, we generated BAP1 knockout (*BAP1*-KO) human mesothelial cell clones to develop molecular-targeted therapeutics based on genetic alterations in MMe. cDNA microarray and quantitative RT-PCR (qRT-PCR) analyses revealed high expression of a *calcium/calmodulin-dependent protein kinase type II subunit delta* (*CAMK2D*) gene in the *BAP1*-KO cells. CAMK2D was highly expressed in 70% of the human MMe tissues (56/80) and correlated with the loss of BAP1 expression, making it a potential diagnostic and therapeutic target for *BAP1*-deficient MMe. We screened an anticancer drugs library using *BAP1*-KO cells and successfully identified a CaMKII inhibitor, KN-93, which displayed a more potent and selective antiproliferative effect against *BAP1*-deficient cells than cisplatin or pemetrexed. KN-93 significantly suppressed the tumor growth in mice xenografted with *BAP1*-deficient MMe cells. This study is the first to provide a potential molecular-targeted therapeutic approach for *BAP1-*deficient MMe.

## Introduction

Malignant mesothelioma (MMe) is a neoplasm caused by exposure to asbestos, erionite, and therapeutic ionizing radiation to the chest [1–4]. It causes ~43,000 deaths worldwide each year [5, 6]. Many MMe cases are resistant to standard chemotherapy and radiation, even after multidisciplinary treatment, including surgical resection. The median survival time after diagnosis in patients with MMe is only 8–12 months. It is an incurable disease with a poor prognosis and outcome [7, 8]. Recent molecular biological studies have revealed frequent genetic alterations in three tumor suppressor genes, *neurofibromatosis 2* (*NF2*), *cyclin-dependent kinase inhibitor 2* *A* (*CDKN2A, p16)*, and *BAP1* in MMe [3, 4, 9, 10]. *BAP1* is also causally linked to a *BAP1* tumor predisposition syndrome, characterized by increased susceptibility to MMe, uveal and cutaneous melanomas, benign melanocytic tumors, and several other cancers [4, 11–14].

Recently, we found high expression of fibroblast growth factor receptor 2 (FGFR2) and CD24 in *NF2*-knockout (KO) mesothelial cell lines [15] and in *NF2/p16*-double knockout (DKO) cell lines, respectively [16], making them potential diagnostic and therapeutic targets for MMe. In this study, we established *BAP1* knockout (*BAP1*-KO) human mesothelial cell clones to develop molecular-targeted therapeutics based on frequent genetic alterations. cDNA microarray and quantitative PCR analyses revealed high expression of a *calcium/calmodulin-dependent protein kinase type II subunit delta* (*CAMK2D*) gene in the *BAP1*-KO cells. Immunohistochemical (IHC) analyses revealed that the loss of BAP1 expression is closely associated with CAMK2D-positive expression in human MMe tissue samples. Ca^2+^ homeostasis is regulated by Ca^2+^/Calmodulin-dependent protein kinase II (CaMKII) in the nerve cells, which is essential not only for the plasticity of the synaptic transmission but also for tumor initiation, angiogenesis, progression, and metastasis [17]. Among the four known CaMKII isozymes, CAMK2D (CaMKII delta) was highly expressed in crocidolite asbestos-exposed lung cell clones [18]. It was also associated with cisplatin resistance in human epithelial ovarian cancer [19]. Together with these reports [17–19], our findings suggest that CAMK2D can be a promising diagnostic and therapeutic target for *BAP1*-deficient MMe. We also screened an anticancer drugs library using *BAP1*-KO cells and identified a CAMKII inhibitor, KN-93 as the most potent cell survival inhibitor. Here, we show that KN-93 exhibits a selective antiproliferative effect against *BAP1*-deficient MMe cells, making it a potential candidate for molecular-targeted anticancer drugs.

## Results

### *BAP1* knockout enhances anchorage-independent cell growth in human mesothelial cells

**Fig. 1:**
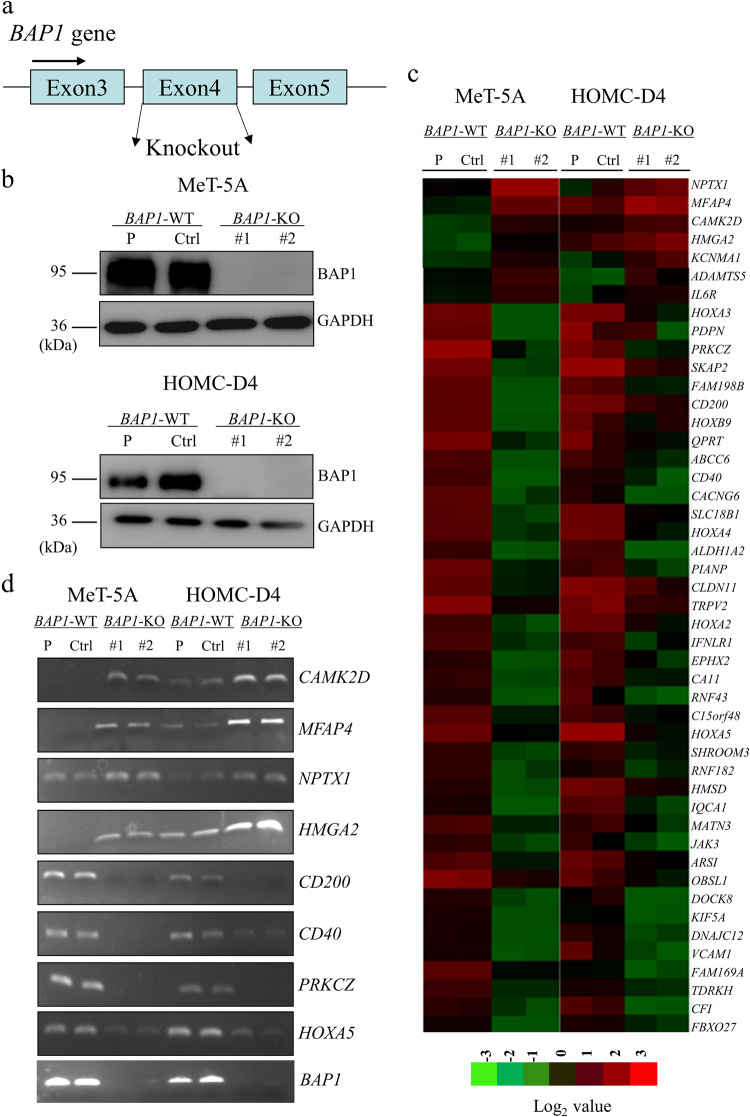
Generation of *BAP1*-KO cell clones and effect of BAP1 loss on the gene expression in human mesothelial cells. **a** Generation of *BAP1*‐KO cell lines using the CRISPR/Cas9 system using a single guide RNA sequence against exon 4 of the *BAP1* gene locus. **b** Establishment of *BAP1*-KO cell clones using human mesothelial cell lines, MeT‐5A and HOMC-D4. BAP1 protein expression was determined by Western blot analysis using GAPDH as the internal control. **c** Gene expression analysis represented by heatmap of upregulated (seven genes; fold change, >3) and downregulated (40 genes, fold change, <0.1) genes in *BAP1*‐KO cells (#1 and #2) compared with parental (P) and *BAP1*-WT (Ctrl) cells. The heatmap was constructed based on the normalized values of all samples using TreeView (Cluster 3.0) http://jtreeview (http://jtreeview.sourceforge.net). The corresponding upregulated or downregulated genes in the heatmap are shown on the right side. **d** RT-PCR analysis for mRNA expression levels of upregulated or downregulated genes in the MeT-5A and HOMC-D4 cells. Representative agarose gels for the RT-PCR products from the parental cells (P), *BAP1*-WT cells (Ctrl), and *BAP1*-KO cell lines (#1 and #2) are shown.

We established two *BAP1*-knockout clones (*BAP1*-KO #1 and #2) using MeT-5A and HOMC-D4 human mesothelial cell lines with the CRISPR/Cas9 system targeting the exon 4 of the *BAP1* gene (Fig. 1a) to develop the molecular-targeted therapeutics based on genetic alterations in MMe. We also randomly selected single MeT-5A/*BAP1* ^+/+^and HOMC-D4/*BAP1* ^+/+^cell clones without the *BAP1* gene knockout as the controls (hereafter called *BAP1*-WT cells, Ctrl). BAP1 expression was not detected in the *BAP1*-KO cells, while it was detectable in the parental and *BAP1*-WT cells (Fig. 1b). The MTT assay revealed no significant change in the growth rate in the *BAP1*‐KO and *BAP1*-WT cells (Fig. S1a). However, in the semi-soft agar colony formation assay, the number of colonies was significantly increased in the *BAP1*-KO cells (Fig. S1b), suggesting that BAP1 loss might enhance anchorage-independent cell growth in human mesothelial cells.

### Gene expression changes induce by *BAP1*-knockout

**Fig. 2:**
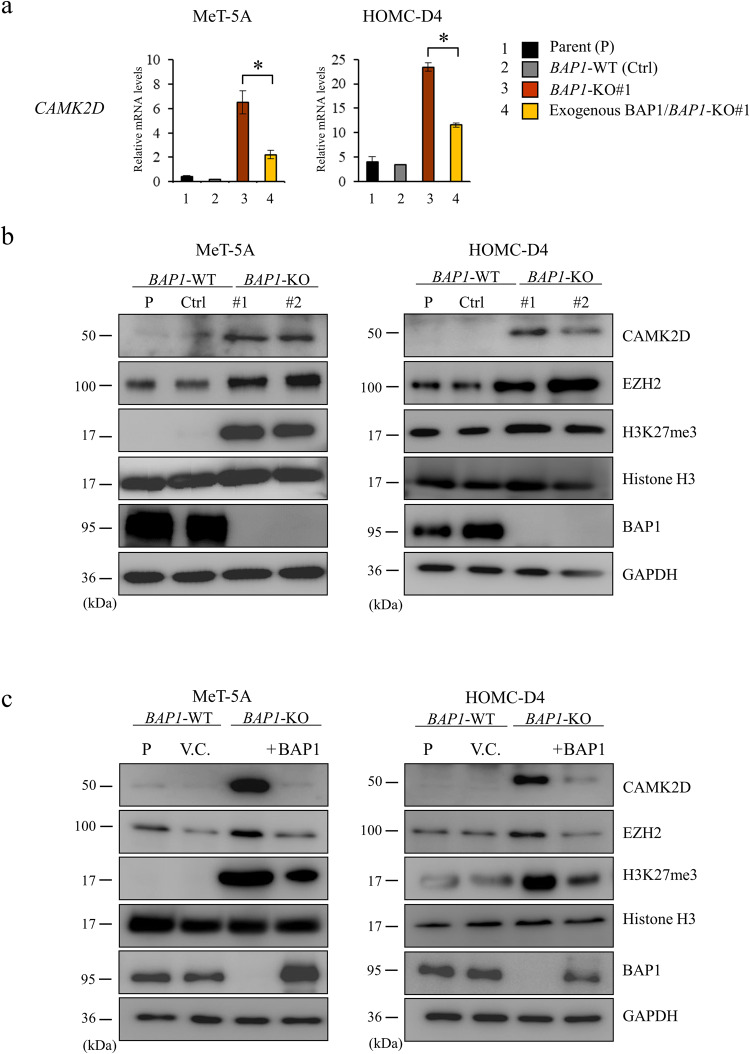
Upregulation of CAMK2D in *BAP1*-KO MMe cells. **a** qRT-PCR analysis of *CAMK2D* using SYBR Green-based method showing the relative gene expression levels after normalization to *GAPDH* expression. The relative transcript levels were calculated by comparing the mean values with those from parental cells. Data are expressed as mean ± SE (*n* = 3). Asterisks indicate significant differences between *BAP1*-KO#1 and exogenous BAP1/*BAP1*-KO#1 cells (*p < 0.05). **b** Western blot analysis for CAMK2D, EZH2, H3K27me3, and BAP1 protein expression in parental (P) and *BAP1*-WT (Ctrl) and *BAP1*‐KO (#1 and #2) cells. **c** Effect of exogenous BAP1 expression on EZH2, H3K27me3, and CAMK2D protein expression in *BAP1*-KO cells. V.C. indicates mock vector control.

We performed cDNA microarray analyses to investigate the differences in the global gene expression profiles in *BAP1*-KO and *BAP1*-WT cells to understand the downstream signaling of BAP1. We successfully detected seven genes with more than 3-fold upregulated expression and 91 genes with less than 0.1-fold downregulated expression in the *BAP1*-KO human mesothelial cells (Fig. 1c and Table S1). Using RT-PCR, we analyzed eight genes to validate the gene expression changes seen in the microarray analysis. Agarose gel electrophoresis of the RT-PCR products showed higher mRNA expression of *CAMK2D*, *MFAP4*, *NPTX1*, and *HMGA2*, but it was lower for *CD200*, *CD40*, *PRKCZ*, and *HOXA5* in the *BAP1*‐KO cells than that in *BAP1*‐WT and parental cells (Fig. 1d). We performed quantitative RT-PCR (qRT-PCR) to precisely compare the expression levels in the cell clones. While the mRNA expression of *CAMK2D*, *MFAP4*, *NPTX1*, and *HMGA2* was significantly increased in *BAP1*-KO cells, it was significantly decreased for *CD200*, *CD40*, *PRKCZ*, and *HOXA5* (Fig. S2). To explore whether BAP1 is involved in these gene expression changes, we exogenously expressed BAP1 in the *BAP1*‐KO cells. Reconstitution of BAP1 into the *BAP1*-KO#1 cells abrogated the expression changes, including upregulation of the *CAMK2D* (Figs. 2a and S3), indicating that CAMK2D might function downstream of BAP1 signaling.

### Loss of *BAP1* expression is associated with histone H3 modification and upregulated CAMK2D expression

BAP1 is a nuclear deubiquitinase that interacts with transcription factors to regulate various cellular pathways, including cell cycle, differentiation, cell death, and gluconeogenesis [20]. LaFave et al. demonstrated that a loss of BAP1 increases the trimethylated histone H3 lysine 27 (H3K27me3) and enhancer of zeste 2 polycomb repressive complex 2 subunits (EZH2) levels [21]. Consistent with this report [21], we observed higher expression of H3K27me3 and EZH2 in the *BAP1*-KO cells than in the parental and *BAP1*-WT cells (Fig. 2b). Additionally, reconstitution of BAP1 into the *BAP1*-KO cells downregulated H3K27me3 and EZH2 (Fig. 2c), suggesting that BAP1 loss might upregulate H3K27me3 and EZH2 and activate their downstream signaling.

A recent study indicated that BAP1 is localized in the endoplasmic reticulum (ER) and bound to the type 3 inositol 1, 4, and 5-triphosphate receptor (IP3R3) [22]. It stabilizes IP3R3 by deubiquitylation, releasing Ca^2+^ from the ER into the cytosol and the mitochondria. Among the seven upregulated genes in *BAP1*-KO cells, we focused on *CAMK2D*, whose protein product regulates Ca^2+^ homeostasis [17]. Western blot analysis showed that CAMK2D expression was markedly higher in the *BAP1*-KO cells than in the parental and *BAP1*-WT cells (Fig. 2b). Additionally, reconstitution of BAP1 into *BAP1*‐KO cells decreased CAMK2D expression (Fig. 2a, c), indicating that the loss of BAP1 might upregulate CAMK2D expression.

### *BAP1* loss is associated with positive CAMK2D expression in human MMe tissues

**Fig. 3:**
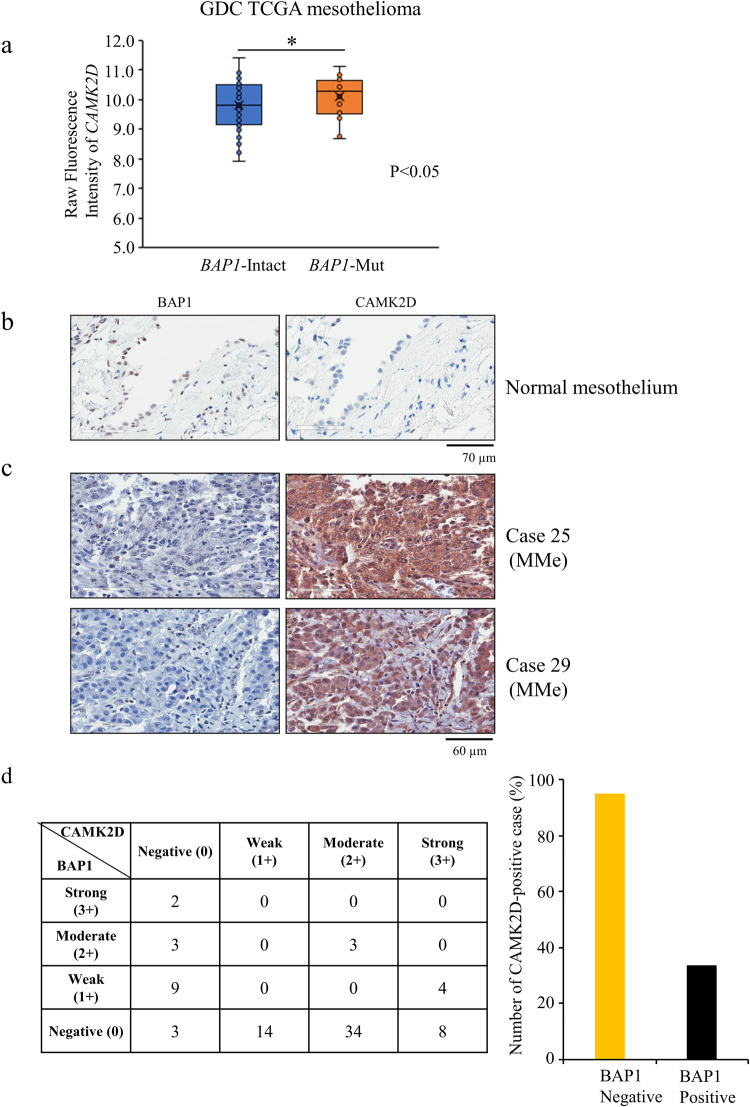
Relationship between BAP1 and CAMK2D expression in MMe tissues. **a** Assessment of *CAMK2D* mRNA expression status in MMe patients with or without *BAP1* mutation using a public database. The raw fluorescence intensity representing *CAMK2D* expression in the specimens from *BAP1*-intact and *BAP1*-mutant MMe patients is shown. A public dataset ID (TCGA-MESO.htseq fpkm-uq.tsv) of specimens from *BAP1*-mutant patients was obtained, formatted, and analyzed. Box and whisker plots of the relative gene expression levels of *CAMK2D* in *BAP1*-intact (*BAP1*-Intact, *n* = 58; blue) and *BAP1*-mutated (*BAP1*-Mut, n = 23; red) MMe patients; the middle line and the error bars indicate the mean and standard deviation, respectively. Asterisks indicate significant differences between *BAP1*-intact and *BAP1*-mutated patients (*P < 0.05). **b**, **c** Representative results of immunohistochemical (IHC) analysis for BAP1 (left panels) and CAMK2D (right panels) expression in a normal mesothelial tissue (**b**) and MMe tissues from cases #25 and #29 (**c**). **d** Summary of IHC results for BAP1 and CAMK2D expression in MMe tissues. Immunoreactivity was independently evaluated by two investigators. Staining intensity was scored as strong (3+), moderate (2+), weak (1+), or negative (0). The bar graph represents the proportion (%) of BAP1-negative or -positive (strong, moderate, and weak) cases with CAMK2D-positive expression (strong, moderate, and weak) in MMe tissues.

To explore the relationship between *BAP1* mutations and *CAMK2D* mRNA expression, we analyzed the publicly available data from the Cancer Genome Atlas (TCGA). We found that the *CAMK2D* expression level in MMe patients with *BAP1* somatic mutations is significantly higher than in patients with intact *BAP1* (Fig. 3a). We next performed IHC analyses of 80 human MMe tissue samples to evaluate CAMK2D expression (Fig. 3b, c and Table S2). Microscopic analysis indicated eight strong (3+), 34 moderate (2+), and 14 weak (1+) CAMK2D-positive signals among these 80 tissue samples (Table S2 and Fig. 3d). Notably, the positivity rate for CAMK2D in BAP1-negative MMe tissues was higher than that in BAP1-positive MMe tissues: 56 of 59 BAP1-negative tissues vs. 7 of 21 BAP1-positive tissues (94% vs. 33%, Fig. 3d). These results indicated that the loss of BAP1 might be closely associated with CAMK2D-positive expression in human MMe tissues.

### CAMKII inhibitor KN-93 selectively suppresses the proliferation of *BAP1*-deficient mesothelioma cells

**Fig. 4:**
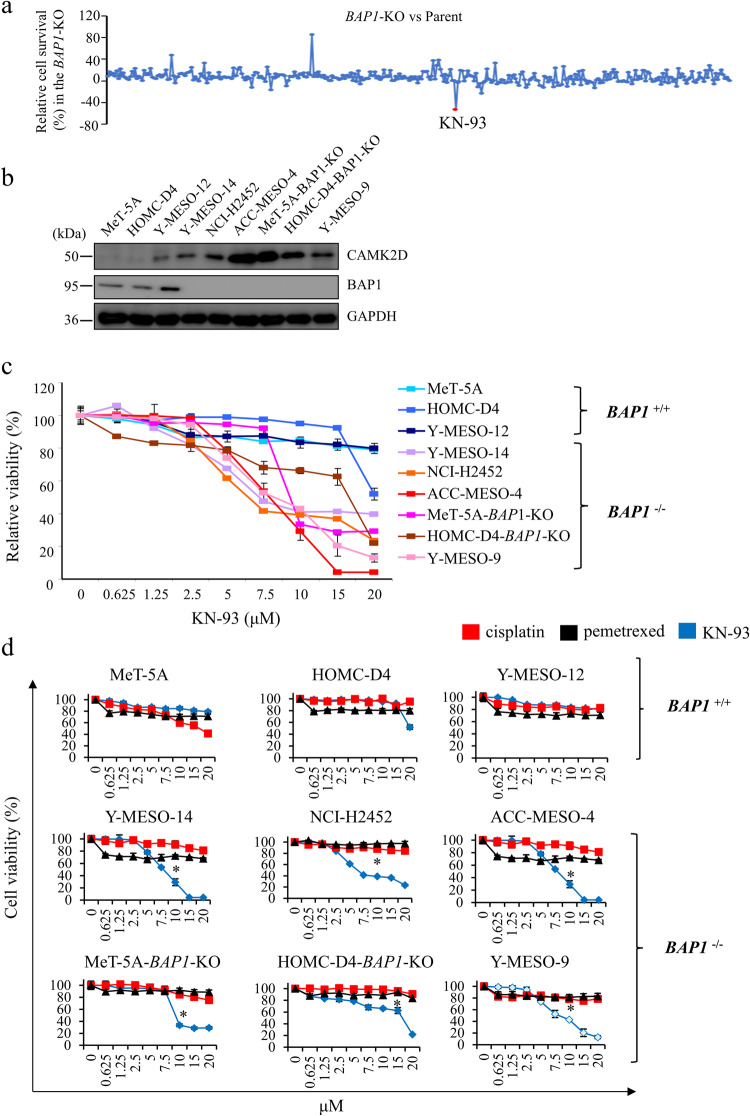
Identification of CaMKII inhibitor KN-93 against *BAP1*-deficient cells and the effect of KN-93 on cell survival of MMe cell lines. **a** Screening assay with the Screening Committee of Anticancer Drugs (SCADS) library. The MeT-5A cells (*BAP1*-KO and parental cells) were treated in the presence or absence of 363 chemical compounds (10 μM each), and the cell survival percentages were calculated after normalization to the mean optical densities in the untreated cells (arbitrarily defined as 100%). The results are shown as differential percentages of cell survival between *BAP1-*WT and *BAP1*-KO cells. The red spot indicates >50% reduction of cell viability in *BAP1*-KO cells compared with *BAP1-*WT cells. **b** Western blot analysis for CAMK2D and BAP1 expression in the mesothelial and MMe cell lines using GAPDH as the internal control. **c** The effect of a CAMKII inhibitor KN-93 on the survival of MMe cell lines. Percentages of cell survival were calculated as described above. **d** The effect of cisplatin, pemetrexed, and KN-93 (Cells were incubated with the indicated concentrations (20, 15, 10, 7.5, 5, 2.5, 1.25, 0.625, and 0 μM) of each drug for 72 h) on the survival of MeT-5A, HOMC-D4, Y-MESO-12, Y-MESO-14, NCI-H2452, ACC-MESO-4, MeT-5A-*BAP1*-KO, HOMC-D4-*BAP1*-KO, and Y-MESO-9 cells. MTT assays were performed according to the manufacturer’s instructions. The absorbance was measured at 595 nm using a spectrophotometer. The cell survival percentages were calculated as described above. Black, red, and blue lines indicate cisplatin, pemetrexed, and KN-93, respectively. Data are expressed as the mean ± SE (*n* = 3). Asterisks indicate significant differences in the efficacy between cisplatin/pemetrexed and KN-93. *p < 0.05.

Since *BAP1* mutation is a frequently occurring genetic alteration in MMe, we explored potential molecular-targeted anticancer drugs for treating MMe with *BAP1* mutations. To this end, we screened the Screening Committee of Anticancer Drugs (SCADS) library, consisting of 363 chemical compounds (http://scads.jfcr.or.jp/kit/index.html), using the *BAP1*-KO MeT-5A and parental cells, and identified a CaMKII inhibitor, KN-93 as the most potent antiproliferative agent (Fig. 4a). The effect of 363 compounds on the cell survival is summarized in Table S3.

We examined the expression of CAMK2D and BAP1 in several MMe cell lines to explore the efficacy of KN-93 on MMe cells. Western blot analysis showed higher expression of CAMK2D in *BAP1*^−/−^cell lines (Y-MESO-14, NCI-H2452, ACC-MESO-4, and Y-MESO-9 along with MeT-5A-*BAP1*-KO, HOMC-D4-*BAP1*-KO cells) than in the *BAP1*^+/+^ cell lines (MeT-5A, parental HOMC-D4, and Y-MESO-12 cells) (Fig. 4b). BAP1 expression was detected only in the parental MeT-5A, parental HOMC-D4, and human mesothelioma Y-MESO12 cell lines (*BAP1*^+/+^) (Fig. 4b). These results, in agreement with the IHC data, indicate that the loss of BAP1 is closely associated with CAMK2D-positive expression in human MMe cell lines.

Subsequently, we assessed the antiproliferative efficacy of KN-93 on several mesothelial and MMe cell lines with or without BAP1 expression. Interestingly, the average IC_50_ value in *BAP1*^+/+^ cells was 20 μM, whereas, in *BAP1*^−/−^ cells, it was 7.5 μM (Fig. 4c). This result suggests that KN-93 selectively suppresses the proliferation of *BAP1*^−/−^ cells with CAMK2D-positive expression. Combination chemotherapy with pemetrexed and cisplatin is relatively ineffective in increasing the survival time of MMe patients. To overcome this, we compared the efficacy of KN-93 with that of cisplatin and pemetrexed using MMe cell lines. We found that KN-93 showed a potent and selective antiproliferative effect against *BAP1*-deficient cells, while cisplatin or pemetrexed did not (Fig. 4d), indicating that KN-93 might be a promising candidate for molecular-targeted anticancer drugs. Therefore, we further compared the antiproliferative effect of KN-93 with the clinically available cisplatin and pemetrexed combination treatment (cisplatin-pemetrexed). The MTT assay showed that cisplatin-pemetrexed does not exert *a* > 50% inhibitory effect on the survival of MMe cells (Fig. S6a). Thus, compared to *BAP1*-WT cells, *BAP1*-deficient cells were more sensitive to treatment with KN-93, whereas there was no difference in the survival rate between *BAP1*-deficient and *BAP1*-WT cells after cisplatin-pemetrexed treatment (Fig. S6b). These results suggested that the clinical use of KN-93 has a benefit regarding antiproliferative action.

### KN-93 induces significant apoptosis in *BAP1*-deficient cells

**Fig. 5:**
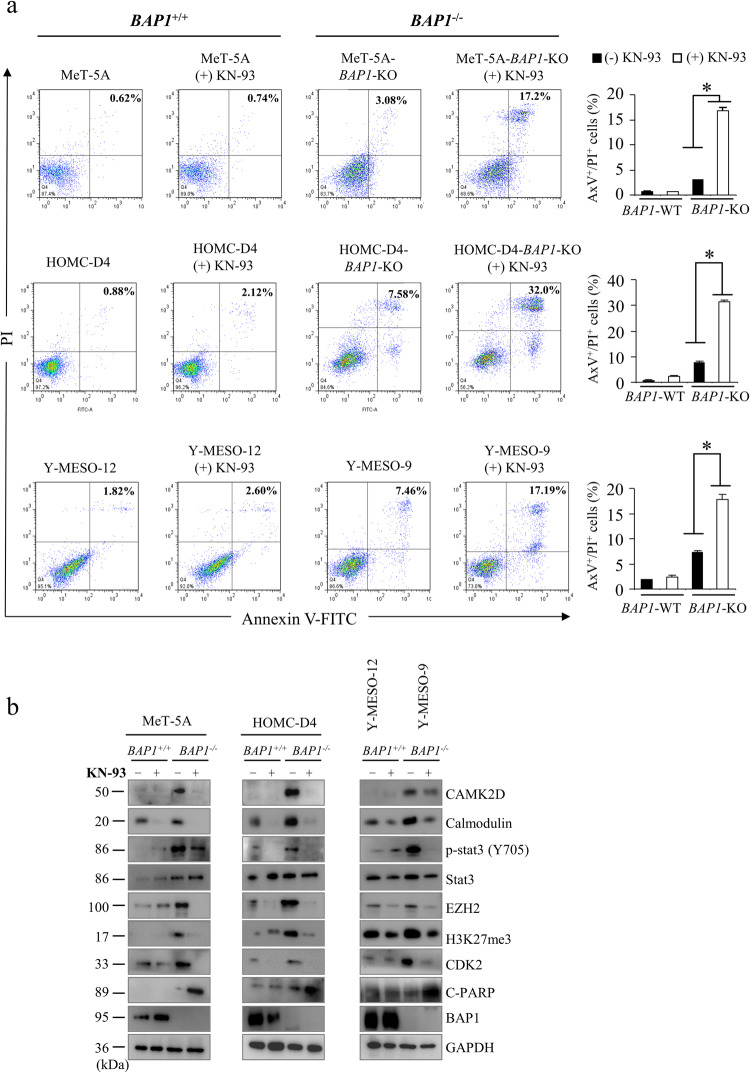
Effect of KN-93 on apoptosis in *BAP1*-deficient MMe cells. **a** Flow cytometry analysis. The representative results of AxV-PI-based staining-based FACS analysis are shown on the left. The graphs on the right show the percentages of AxV^+^/PI^+^ apoptotic cells after treating the cells with KN-93 (7.5 µM) for 48 h, measured using FACS CantoII. Data are expressed as the mean ± SE (*n* = 3). Asterisks indicate significant differences between *BAP1*-deficient cells (*BAP1*-KO Met-5A and HOMC-D4 cells and Y-MESO-9 cells). *p < 0.05. **b** Western blot analysis for BAP1, CAMK2D, calmodulin, p-STAT3, STAT3, EZH2, H3k27me3, CDK2, and c-PARP protein expression in MeT-5A, MeT-5A-*BAP1*-KO, HOMC-D4, HOMC-D4-*BAP1-KO*, Y-MESO-12, and Y-MESO-9 cells treated with KN-93 (7.5 µM) for 48 h.

We performed annexin V (AxV)/propidium iodide (PI) double staining-based FACS analysis to examine the effect of KN-93 on apoptosis. The number of AxV^+^/PI^+^ cells was significantly higher in the KN-93-treated *BAP1*^−/−^ (MeT-5A-*BAP1*-KO, HOMC-D4-*BAP1*-KO, and Y-MESO-9) cells than in the *BAP1*^+/+^ (parental MeT-5A, parental HOMC-D4, and Y-MESO-12) cells (Fig. 5a). Similarly, the cleaved PARP level was markedly increased in the KN-93-treated *BAP1*^*–/–*^cells (Fig. 5b), suggesting that KN-93 suppresses the proliferation of *BAP1*^−/−^cells by promoting apoptosis. Consistently, the expression of CAMK2D, calmodulin (CaM), phospho-STAT3 (Y705), EZH2, H3K27Me3, and CDK2 was also decreased in the *BAP1*^−/−^cells (Fig. 5b). Additionally, we generated *BAP1*/*CAMK2D*-double knockout (DKO) cells to further investigate the involvement of CAMK2D in the proliferations of the *BAP1*^−/−^cells (Fig. S4a). We found that *CAMK2D*/*BAP1*-DKO reduced cell proliferation, strongly suggesting that CAMK2D might play a critical role in the tumor growth of *BAP1*-deficient MMe cells (Fig. S4b).

We next measured the intracellular Ca^2+^ concentrations in the HOMC-D4 cell clones to study the effect of the CAMKII inhibitor KN-93 on Ca^2+^ homeostasis. The Ca^2+^ levels in *BAP1*-KO cells were significantly lower, while the levels in the *BAP1*/*CAMK2D*-DKO cells were similar to those in the parental cells (Fig. S4c). KN-93 treatment significantly increased the intracellular Ca^2+^ levels in the *BAP1*^−/−^cells but not in the parental cells (Fig. S4d). We further measured intracellular Ca^2+^ concentrations in four MMe cell lines. The Ca^2+^ levels was significantly lower in *BAP1*^−/−^ (Y-MESO-9 and Y-MESO-14) cells than those in the *BAP1*^+/+^ (Y-MESO-12, and MSTO-211H) cells (Fig. S4e). Again, KN-93 treatment significantly increased intracellular Ca^2+^ levels in the *BAP1*^*–/–*^ MMe cells but not in the *BAP1*^+/+^ MMe cells (Fig. S4e). Based on these results, we propose that KN-93 can promote apoptosis by increasing intracellular Ca^2+^ concentrations, most likely by inhibiting CAMK2D activity in the *BAP1*^−/−^ HOMC-D4 and MMe cells.

### KN-93 suppresses the tumor growth of Y-MESO-9 in xenografted mice

**Fig. 6:**
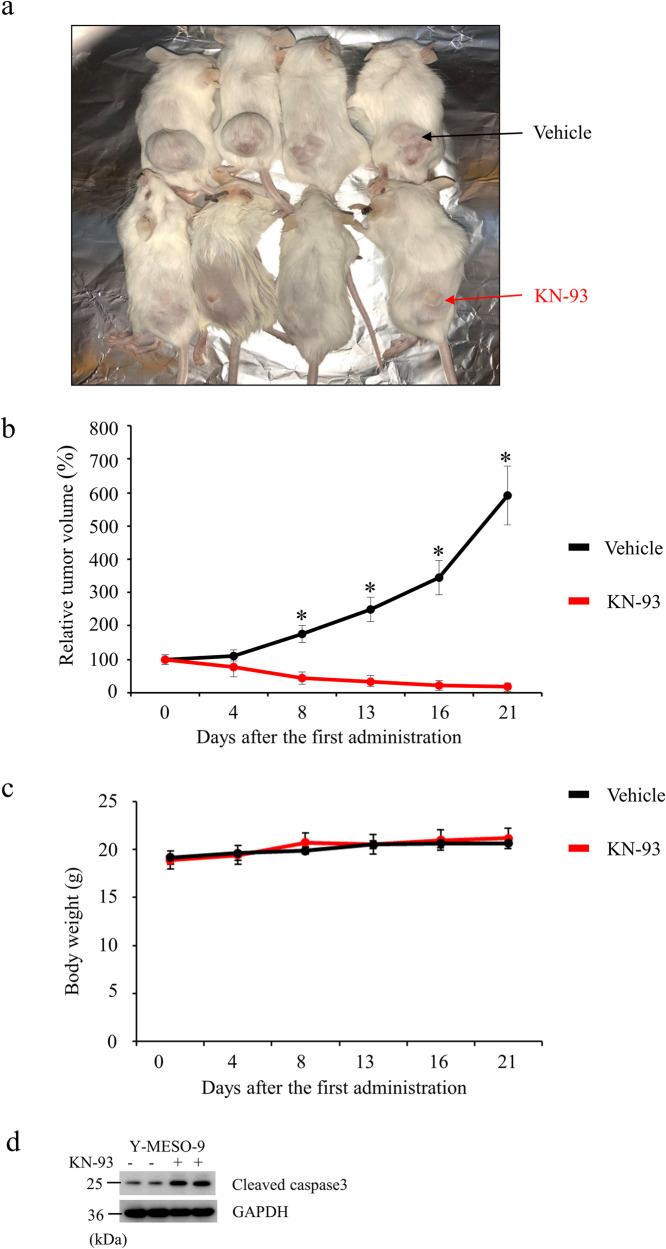
Effect of KN-93 on the tumor growth of Y-MESO-9 cells in vivo. Y-MESO-9 cells (*BAP1*^−/−^, 1 × 10^7^ cells/mouse) were subcutaneously xenografted into SCID mice. After the tumor volume reached 100 mm^3^ (day 0), KN-93 (15 mg/kg body weight) or vehicle (PBS) was intraperitoneally administered on days 0, 4, 8,13, and 16 into xenografted mice. **a** A representative picture of tumor-bearing xenografted mice in each group is shown. **b**, **c** Line graphs show (**b**) the relative tumor volume and (**c**) the body weight of mice during the treatment with KN-93. The tumor volume is expressed relative to the tumor size at day 0, arbitrarily defined as 100%. Data are expressed as the mean ± SE (*n* = 4).). *p < 0.05. **d** Western blot analysis using cleaved caspase3 showing the efficacy of KN-93 in tumor.

We examined the in vivo effect of KN-93 on the tumor growth of *BAP1*^−/−^cells (Y-MESO-9) using xenografted mice. KN-93 administration to mice significantly suppressed the tumor growth compared with vehicle control (Fig. 6a, b) without causing weight loss (Fig. 6c). These findings further support that targeting CAMK2D using KN-93 might be a viable therapeutic approach for *BAP1*-deficient MMe. Moreover, the cleaved caspase-3 level markedly increased in KN-93-treated tumors (Fig. 6d), suggesting that KN-93 suppresses tumor growth at least in part by promoting apoptosis. In addition, we did not observe any death in KN-93-treated and/or vehicle control groups (data not shown). To further investigate the effects of KN-93 on *BAP1*-WT MMe cells, we performed a xenograft experiment with MSTO-211H (*BAP1*-WT) cells. KN-93 administration did not reduce the tumor growth of *BAP1*-WT MSTO-211H cells (Fig. S7).

### KN-93 administration does not affect body weight, liver, and renal functions

Finally, we evaluated the in vivo adverse effects of KN-93 on normal BALB/c strain mice. KN-93 administration did not cause significant changes in histological images of the heart, liver, and kidney (Fig. S5a) or body weight of mice (Fig. S5b) during the 14-day study period. Additionally, KN-93 administration did not significantly change blood chemical data (Table S6). Thus, our histochemical and blood chemical analyses did not reveal any obvious impairment of heart, liver, and renal functions in KN-93-treated mice. These results support our notion that KN-93 is a promising candidate for molecular-targeted anticancer drugs for treating *BAP1*-deficient MMe.

## Discussion

*BAP1*, a major tumor suppressor gene, frequently undergoes genetic mutations in MMe. We established *BAP1*-KO isogenic clones using immortalized human mesothelial cell lines to develop molecular-targeted therapeutics based on genetic alterations in MMe. cDNA microarray analysis revealed that several genes were upregulated or downregulated in the *BAP1*-KO cells. Based on recent evidence showing the involvement of BAP1 in intracellular Ca^2+^ homeostasis [22], we focused on *CAMK2D* out of the seven upregulated genes. CAMK2D is one of four CaMKII isozymes, typically activated by binding to Ca^2+^/CaM via its regulatory domain and a multifunctional protein kinase that transmits calcium signaling [17, 23, 24]. Several studies have shown high CaMKII expression in lung, breast, colon, and prostate cancers [25–28]. Our molecular biology and IHC studies revealed high CAMK2D expression in the BAP1-negative MMe tissues and cell lines, indicating that BAP1 loss might be linked with CAMK2D-positive expression. Thus, it is conceivable that *BAP1* deficiency might participate in the tumorigenesis of mesothelial cells by mediating CAMK2D expression.

In this study, we also found that gene expression levels of *MFAP4*, *NPTX1*, and *HMGA2* significantly increased in *BAP1*-deficient human mesothelial cells. Thus, it would be of particular interest to examine whether knockdown or inhibition of MFAP4, NPTX1, and/or HMGA2 suppresses tumor development of *BAP1*-deficient MMe cells.

Since BAP1 regulates Ca^2+^ homeostasis [22, 29], we evaluated the intracellular Ca^2+^ levels and observed lower levels in *BAP1*-KO cells than those in parental cells. However, the Ca^2+^ levels in the *CAMK2D*/*BAP1*-DKO cells were restored to those similar to parental cells, suggesting that CAMK2D might regulate the intracellular Ca^2+^ levels in the *BAP1*-deficient cells. Moreover, *BAP1*-KO had a negligible effect on cell proliferation, whereas *CAMK2D*/*BAP1*-DKO reduced cell proliferation, suggesting that an emerging BAP1-CAMK2D axis might play a critical role in the tumor growth of *BAP1*-deficient MMe cells. Our findings support that CAMK2D is a potential diagnostic and therapeutic target for *BAP1*-deficient MMe.

Upon screening an anticancer drugs library with *BAP1*-KO cells, we successfully identified a CaMKII inhibitor, KN-93 as the most potent cell survival inhibitor. KN-93 inhibits CaMKII activity by interfering with its binding to the Ca^2+^CaM complex [30, 31]. After identifying KN-93, we investigated its inhibitory effect on several BAP1-positive and negative MMe cell lines. We found that it selectively suppressed the proliferation of BAP1-negative cells rather than BAP1-positive cells. The median overall survival of MMe patients after frontline chemotherapy with pemetrexed and cisplatin is ~12 months only [7, 32]. Novel therapies are urgently needed to improve the prognosis of MMe patients. Therefore, we further compared the efficacy of KN-93 with both pemetrexed and cisplatin in MMe cells. KN-93 exhibited a robust and selective antiproliferative effect against BAP1-negative MMe cells, while pemetrexed or cisplatin did not.

Then, we examined the effect of KN-93 on the apoptosis of MMe cell lines and observed that it significantly increased DNA damage response molecules PARP, thus inducing apoptosis in BAP1-negative cells. Additionally, KN-93 markedly increased the intracellular Ca^2+^ levels in BAP1-negative cells but not in parental cells. Hence, we propose that KN-93 promotes apoptosis in BAP1-negative MMe cells by increasing intracellular Ca^2+^ concentrations, most likely by inhibiting CAMK2D activity. Previously, a Ca^2+^-regulated decrease in apoptosis has been implicated in the cellular transformation in BAP1-related cancers [22].

Finally, we examined the effect of KN-93 on the tumor growth of *BAP1*-negative MMe cells in vivo. We administered KN-93 to SCID mice xenografted with *BAP1*-deficient MMe cells. We observed significantly lower tumor volume in these mice than in the vehicle control without any effect on the body weight. Moreover, KN-93 was shown to have no significant adverse impact on the histochemistry of heart, liver, and kidney or blood chemistry in normal BALB/c strain mice. All these results strongly suggest a lack of apparent toxic side effects. Based on these findings, we advocate that KN-93 is a promising molecular-targeted anticancer drug for treating *BAP1*-deficient MMe.

In this study, we showed that CAMK2D expression was readily increased in *BAP1*-knockout (*BAP1*-KO) immortalized human mesothelial cell lines MeT-5A and HOMC-D4, in which artificial SV40, and/or hTERT gene expression were transduced; we did not confirm whether SV40 or hTERT gene expression affects CAMK2D expression in MMe cells. Although our IHC analysis showed that the loss of the *BAP1* signal is closely associated with increased CAMK2D expression in MMe tissues, further functional experiments (e.g., gene knockdown and/or knockout) should be performed to confirm whether the loss of *BAP1* leads to CAMK2D upregulation in MMe cells.

In summary, our cDNA microarray and qRT-PCR analyses revealed high expression of *CAMK2D* in the *BAP1*-deficient mesothelial cells. Subsequent molecular biology and IHC studies revealed that the loss of BAP1 is closely associated with CAMK2D-positive expression in human MMe tissues and cell lines. Although the molecular mechanism underlying CAMK2D upregulation via the loss of BAP1 remains unclear, *BAP1* deficiency might be involved in tumorigenesis by mediating CAMK2D expression. Therefore, CAMK2D might be a novel diagnostic and therapeutic target for MMe. Moreover, we identified a CAMKII inhibitor KN-93 as a potent and selective chemical antiproliferative agent, making it a potential molecular-targeted anticancer drug for treating *BAP1*-deficient MMe. Previous studies reported a significantly high incidence (more than 60%) of MMe with *BAP1* alterations [4, 33, 34]. Therefore, targeting the BAP1-CAMK2D (CAMKII delta) signaling axis is a promising therapeutic approach for most MMe cases and *BAP1* tumor predisposition syndrome.

## Materials and methods

### Cell culture

Two immortalized normal human mesothelial cell lines, MeT-5A (pleural mesothelial) and HOMC-D4 (omental mesothelial; intermediate type), and five human mesothelioma cell lines, ACC-MESO-4, Y-MESO-12, Y-MESO-14, Y-MESO-9, and NCI-H2452, were kindly provided by Dr. Y. Sekido, Division of Molecular Oncology, Aichi Cancer Center Research Institute (Nagoya, Japan). The HOMC-D4 cell line was maintained as described previously [35]. Y-MESO-9, Y-MESO-12, Y-MESO-14, ACC-MESO-4, MSTO-211H, and NCI-H2452 cell lines were cultured in RPMI-1640 (Wako, Osaka, Japan) medium containing 10% fetal bovine serum (Sigma) and 1% penicillin-streptomycin (Wako) at 37 °C in a 5% CO_2_ humidified atmosphere.

### Gene knockout using the clustered regularly interspaced short palindromic repeat (CRISPR)/Cas9 system

The CRISPR/Cas9 system was used to disrupt the *BAP1* gene as described previously [36, 37]. pSpCas9(BB)-2A-GFP (PX458) was gifted by Feng Zhang (Addgene plasmid # 48138) [36]. Briefly, a single guide RNA (sgRNA) sequence was selected using an optimized CRISPR design (http://crispr.mit.edu/). The sgRNA sequence used for *BAP1* was 5′- TCAAATGGATCGAAGAGCGC -3′ and the sequence used for *CAMK2D* was 5′- CACCGCAGCATATTCTTGTCCAGT -3′, corresponding to exons 4 and 2, respectively. The plasmid expressing hCas9 and the sgRNA were prepared by ligating the oligonucleotides into the *BbsI* site of PX458 (*BAP1*/PX458 and *CAMK2D*/PX458).

The knockout clones were created by electroporating 1 μg of *BAP1*/PX458 plasmid into 1 × 10^6^ cells using a 4D-Nucleofector™ system (Lonza Japan, Tokyo, Japan). Three days post-transfection, GFP-expressing cells were sorted using BD FACS ARIA III (BD Bioscience). A single clone was selected, expanded, and used for biological assays. Cells disrupting the *BAP1* gene and losing its protein expression were used as *BAP1*-KO clones, whereas cells expressing BAP1 without any mutations on the *BAP1* gene locus were used as *BAP*1-WT controls.

For BAP1 transduction, the *BAP1*/pcDNA3.1 vector was transfected into a *BAP1*-KO#1 clone with the 4D-Nucleofector System using the backbone pcDNA3.1 as a control vector. After transfection, the cells were incubated for 48 h, washed with phosphate-buffered saline (PBS), and lysed in the loading buffer. The lysates were used for Western blot analysis to check BAP1 expression.

### Quantitative RT-PCR

qRT-PCR analysis was performed using SYBR Green I detection as previously described [38] using *glyceraldehyde-3-phosphate dehydrogenase* (*GAPDH*) as the internal control. The primers used are provided in Table S4.

### cDNA microarray analysis

cDNA microarray analysis was conducted based on the manufacturer’s protocol (Agilent Technologies), as described previously [39]. Briefly, we performed cDNA synthesis and cRNA labeling with cyanine 3 (Cy3) dye using the Agilent Low Input Quick Amp Labeling Kit (Agilent Technologies). The Cy3-labeled cRNA was purified, fragmented, and hybridized onto a Human Gene Expression 8x60K v2 Microarray Chip containing 62,969 Entrez Gene RNAs using a Gene Expression Hybridization kit (Agilent Technologies). The raw and normalized microarray data were submitted to the NCBI GEO database (accession number GSE168340).

### Cell growth assay

The cell growth rate was measured using MTT assays. Briefly, 1 × 10^3^ cells/well were seeded into 96-well plates and cultured for the indicated times. Subsequently, 10 μL of MTT solution (5 mg/mL; Sigma-Aldrich) was added to each well, and the cells were incubated for 4 h. Next, cell lysis buffer was added to the wells to dissolve the colored formazan crystals. The absorbance was measured at 595 nm using a SpectraMAX M5 spectrophotometer (Molecular Devices, Sunnyvale, CA, USA).

### Soft agar colony formation assay

A soft agar colony formation assay was performed as described previously [15, 16, 40]. The number of colonies was counted using Colony Counter software (Keyence, Tokyo, Japan).

### Western blot analysis

Western blot analysis was performed as described previously [41]. The antibodies used are listed in Table S5. Immune complexes were detected using Immuno Star LD (Wako Pure Chemical Industries, Ltd., Osaka, Japan) along with a LAS-4000 image analyzer (GE Healthcare, Tokyo, Japan).

### Annexin V assay

Cells were seeded into 6-well culture plates (5 × 10^5^ cells/ well) and incubated with KN-93 (7.5 µM) for 48 h. After incubation with annexin V (Ax)-FITC and PI (10 μg/mL) at room temperature for 15 min, the fluorescence intensities were measured using fluorescence-activated cell sorting (FACS) using a FACS CantoII (BD, Franklin Lakes, NJ, USA).

### Immunohistochemistry

IHC analysis was performed based on a previous method [41] using a human mesothelioma tissue array from US Biomax (MS801b and MS-1001a; Rockville, MD, USA). Sections were incubated with primary antibodies (BAP1 and CAMK2D antibodies, 2 μg/mL). We used either normal rabbit immunoglobulin G or no primary antibody as negative controls. Immunoreactivity was evaluated independently by two investigators (S.K. and H.M.). The staining intensity was scored as strong (+3), moderate (+2), weak (+1), or negative (0).

### Screening of anticancer drugs library

The Screening Committee of Anticancer Drugs (SCADS) library, containing 363 compounds in four 96-well microplates (http://scads.jfcr.or.jp/kit/index.html) was kindly provided by Grant-in-Aid for Scientific Research on Innovative Areas, Scientific Support Programs for Cancer Research, from The Ministry of Education, Culture, Sports, Science and Technology, Japan. The compounds, consisting mostly of anticancer drugs and kinase inhibitors, were originally dissolved at a concentration of 10 mM in dimethyl sulfoxide (DMSO) solution. *BAP1*-KO and parental MeT-5A cells (3 × 10^3^ cells /well) were seeded into 96-well plates. Next day, cells were treated with a final concentration of 10 μM of each compound or diluent DMSO control (final 0.1% DMSO), and were further incubated for 72 h. MTT assays were performed according to the manufacturer’s instructions. The absorbance was measured at 595 nm using a spectrophotometer. The percentages of cell survival are calculated after normalizing to the mean optical densities in the untreated cells, which were arbitrarily defined as 100%. To identify the compounds with potent antiproliferative effects on *BAP1*-KO cells, we calculated the differences in the cell survival percentages between *BAP1*-KO and parental cells.

### Xenograft experiments

The animal experiments were approved by the ethics committee of Aichi Medical University and performed according to the established guidelines. Female Fox Chase SCID mice (CB17/Icr-Prkdcscid/IcrIcoCrl) (6–8 weeks old, each weighing 17–18 g) were purchased from CLEA Japan, Inc (Tokyo, Japan) and bred at the Institute of Animal Experiments in Aichi Medical University in specified pathogen-free animal facilities. For xenografting, 1 × 10^7^ Y-MESO-9 cells in 80 μL phosphate-buffered saline were mixed with 20 µL Matrigel (phenol red-free; Corning) on ice. The mixture was subcutaneously injected into the back of mice using a 27 G needle [35]. When the inoculated tumor reached 100 mm^3^ (day 0), the mice were randomly divided into two groups (treatment and control groups). KN-93 (15 mg/kg body weight) was intraperitoneally administered on days 0, 4, 8, 13, and 16 to each mouse in the treatment group. The control group received PBS as vehicle control. The tumor volume was measured every three to four days and calculated using the modified ellipsoid formula (1/2 × length × width^2^).

### In vivo effects of KN-93 on BALB/c mice

Eight-week-old female BALB/cCrSlc (*n* = 5, Japan, Inc, Tokyo, Japan) mice were obtained to evaluate the in vivo effects of KN-93. On days 0, 3, 5, 7, 10, and 12, KN-93 (15 mg/kg body weight) or PBS (vehicle control) was intraperitoneally administered to mice. Body weights were measured three times a week. At 14 days after the first administration, the control and treatment group mice were anesthetized using isoflurane, and ~0.5–1 mL of blood was collected in a heparin tube. The heparin-plasma samples were examined for blood chemistry by the Nagahama Life Science Laboratory (Oriental Yeast Co., Ltd., Shiga, Japan). The heart, liver, and kidney were carefully dissected for histochemical analyses from mice after the isolation of whole blood. Each organ was washed with PBS and fixed with phosphate-buffered formalin for 24 h. The tissues were trimmed, embedded in paraffin, sectionized into 5-µm thick sections, and stained with hematoxylin and eosin (H&E) (ab245880, Abcam, Cambridge, UK). The stained sections were observed by a microscope (BX41, Olympus, Tokyo, Japan).

### Intracellular Ca^2+^ assay

Intracellular Ca^2+^ levels were measured using the CS22 Calcium kit-Fluo 4 (Dojindo, Japan) according to the manufacturer’s protocol. Cells were seeded into 96-well culture plates (2 × 10^5^ cells/ well) and incubated with KN-93 (7.5 µM) for 12 h. Shortly after removing the medium without damaging the cells, 100 μL of loading buffer was added to each well of 96-well plates. After incubating the cells at 37 °C for 1 h, the loading buffer was replaced with 100 μL warm recording medium (1X) preheated to 37 °C. Fluorescence intensity changes were measured at an excitation wavelength of 480–500 nm and emission at 518 nm.

### Statistical analysis

The results are expressed as the mean ± SE. Statistical significance between groups was determined using a one-way analysis of variance and Dunnett’s comparison. Statistical analyses were performed using SPSS 23.0 software (SPSS Inc, Chicago, IL, USA).

## Supplementary information

Supplementary Figure legends

Table S1. Upregulated or downregulated genes in the BAP1-KO clones

Table S2. Summary of immunohistochemistry in this study

Table S3. Effect of 363 compounds on the proliferation of BAP1-KO and Parental Met-5A cells

Table S4. Primer sets used in this study

Table S5. Antibodies used in this study

Table S6. Blood chemistry in vehicle control mice and KN-93-treated mice

Fig. S1.Effect of BAP1 loss on the proliferation and colony formation in MeT-5A and HOMC-D4 cells

Fig. S2. Effect of BAP1 loss on the gene expression in MeT-5A and HOMC-D4 cells

Fig. S3. Effect of exogenous BAP1 on the gene expression

Fig. S4. Effect of BAP1 loss and KN-93 treatment on intracellular Ca2+ levels

Fig. S5. In vivo effects of KN-93 on BALB/cCrSlc mice

Fig. S6. Effect of cisplatin and pemetrexed combination treatment on cell viability

Fig. S7. Effect of KN-93 on the tumor growth of MSTO-211H cells in vivo

Fig. S8. mRNA expression of BAP1 in MeT-5A and HOMC-D4 cells

Full and uncropped western blots images

## Data Availability

All data are available in the main text or the supplementary material. Also available based on reasonable request to corresponding authors.
