# Supplementary material for: CAMK2D: a novel molecular target for BAP1-deficient malignant mesothelioma
Source: Cell Death Discov. 2023 Jul 21;9:257. doi: 10.1038/s41420-023-01552-5 (PMC10362017; doi:10.1038/s41420-023-01552-5)
Supplement: Supplementary file 1 — Supplementary Figure legends [file 41420_2023_1552_MOESM1_ESM.docx]

**Supplementary legends**

**Fig S1**. **Effect of BAP1 loss on the proliferation and colony formation in** **MeT-5A and HOMC-D4 cells**

(**a**) MTT assay results show the percentages of cell survival in parental cells (P), *BAP1* WT cells (Ctrl), and *BAP1*-KO on days 0, 3, 5, and 7 post-incubation. Data are the mean ± SE (n = 3). (**b**) Representative soft agar colony formation assays are shown. The bar graphs on the right represent the number of stained colonies. Scale bar = 100 μm. Data are expressed as mean ± SE (n = 3). **p* < 0.05; statistically significant difference between *BAP1*-WT and *BAP1*-KO cells.

**Fig. S2.** **Effect of BAP1 loss on the gene expression in MeT-5A and HOMC-D4 cells**

mRNA expression levels of upregulated genes (**a, b**) or downregulated genes (**c, d**) in parental cells, *BAP1* WT cells (Ctrl), and *BAP1*-KO#1 and #2 cells were quantitated using qPCR analyses. Relative gene expression levels are shown after normalization to *GAPDH* mRNA expression. Mean values were compared with the normal control value to calculate the relative amounts of the transcripts. Data are expressed as mean ± SE (n = 3). Asterisks indicate significant differences between *BAP1*-WT and *BAP1*-KO cells. **p* < 0.05.

**Fig. S3. Effect of exogenous BAP1 on the gene expression**

(**a-d**) qPCR analysis. (**a-b**) Expression levels of *MFAP4*, *NPTX1*, and *HMGA2* genes in parental cells, *BAP1*-WT cells (Ctrl), *BAP1*-KO#1 clone, and *BAP1*-KO#1 clone exogenously expressing BAP1in MeT-5A (a) and HOMC-D4 cells (b). (**c-d**) Expression levels of *CD200*, *CD40*, *PRKCZ*, and *HOXA5* *MFAP4*, *NPTX1*, and *HMGA2* genes in parental cells, *BAP1*-WT cells (Ctrl), *BAP1*-KO#1 cells, and *BAP1*-KO#1 cells exogenously expressing BAP1 in MeT-5A (c) and HOMC-D4 cells (d). The relative gene expression levels are shown after normalization to *GAPDH* expression. The mean values were compared with normal control values to calculate the relative transcript levels. Data are expressed as the mean ± SE (n = 3). Asterisks indicate significant differences between *BAP1*-KO#1 and *BAP1*-KO#1 cells exogenously expressing BAP1. * *p* < 0.05.

**Fig. S4. Effect of BAP1 loss and KN-93 treatment on intracellular Ca^2+^ levels**

(**a**) Generation of *BAP1/CAMK2D*-DKO cell clones. (**b**) The effect of *BAP1*-KO and *BAP1/CAMK2D*-DKO on the proliferation in HOMC-D4 cells. (**c**) The effect of *BAP1*-KO and *BAP1/CAMK2D*-DKO on intracellular Ca^2+^ levels in HOMC-D4 cells. Relative intracellular Ca^2+^ levels are shown after normalization to parental cells, arbitrarily regarded as 1. (d) The effect of KN-93 on intracellular Ca^2+^ levels in parental, *BAP1*-KO#1 and *BAP1*-KO#2 HOMC-D4 cells. (**e**) The effects of *BAP1*^–/–^ and KN-93 on intracellular Ca^2+^ levels in Y-MESO-12, MSTO-211H, Y-MESO-9 and Y-MESO-14 MMe cells. Data are expressed as the mean ± SE (n = 3). **p* < 0.05

**Fig. S5. *In vivo* effects of KN-93 on BALB/cCrSlc mice**

(**a**) Representative results of histochemical images of the heart, liver, and kidney from mice after the 14-day study period (H&E staining, magnification, ×100; scale bar = 100 μm). (**b**) Body weight changes in mice during the 14-day study period

**Fig. S6. Effect of cisplatin and pemetrexed combination treatment on cell viability.**

(**a**) MeT-5A, HOMC-D4, Y-MESO-12, Y-MESO-14, NCI-H2452, ACC-MESO-4, MeT-5A-BAP1-KO, HOMC-D4-BAP1-KO, and Y-MESO-9 cells were seeded in a 96-well plate (cell density, 3 × 10^3^ cells/well). The following day, cells were treated with cisplatin and pemetrexed combination (at concentrations of 20, 15, 10, 7.5, 5, 2.5, 1.25, 0.625, and 0 μM) for 72 h. MTT assays were performed according to the manufacturer’s instructions. Cell survival percentages were calculated as described above. Absorbance was measured at 595 nm using a spectrophotometer, which was arbitrarily defined as 100%. Data are the mean ± SE (n = 3). (**b**) Cell survival was compared to cisplatin and pemetrexed (combination 20 μM) and KN-93 (20 μM) in *BAP1*^+/+^ and *BAP1*^-/-^ cells. Graphs show the percentages of cell viability after treating cells with KN-93 (20 µM). Data are the mean ± SE (n = 3). Asterisks indicate significant differences between KN-93 and combination (cisplatin and pemetrexed) treatment in *BAP1*-deficient cells (*p < 0.05).

**Fig. S7. Effect of KN-93 on the tumor growth of MSTO-211H cells *in vivo*.**

MSTO-211H cells (*BAP1*^+/+^; 5 × 10^6^ cells/mouse) were subcutaneously xenografted into SCID mice. After the tumor volume reached 100 mm^3^ (day 0), KN-93 (15 mg/kg body weight) or vehicle (PBS) was intraperitoneally administered on days 0, 3, 5, 8, and 11 into xenografted mice. (a) Representative picture of tumor-bearing xenografted tumors in each group. (b and c) Line graphs of the relative tumor volume (b) and body weight (c) of mice during treatment with KN-93. The tumor volume was expressed relative to the tumor size on day 0, arbitrarily defined as 100%. Data are the mean ± SE (n = 6).

**Fig. S8. mRNA expression of *BAP1* in MeT-5A and HOMC-D4 cells.**

Parental cells, *BAP1*-WT cells (Ctrl), and *BAP1*-KO#1 and #2 cells were quantitated using qPCR analyses in MeT-5A (a) and HOMC-D4 (b) cells. Relative gene expression levels are shown after normalization to GAPDH mRNA expression. Mean values were compared to the normal control value to calculate the relative amounts of the transcripts. Data are mean ± SE (n = 3).
