# Supplementary material for: CAMK2D: a novel molecular target for BAP1-deficient malignant mesothelioma
Source: Cell Death Discov. 2023 Jul 21;9:257. doi: 10.1038/s41420-023-01552-5 (PMC10362017; doi:10.1038/s41420-023-01552-5)
Supplement: Supplementary file 2 — Table S1. Upregulated or downregulated genes in the BAP1-KO clones [file 41420_2023_1552_MOESM2_ESM.docx]

Table S1. Upregulated or downregulated genes in the *BAP1*-KO clones

| No. | Gene symbol | Description | MeT-5A-*BAP1*-KO vs parent | HOMC-A4-*BAP1*-KO vs parent |
| --- | --- | --- | --- | --- |
|  | Upregulated genes |  | Fold change | Fold  change |
| 1 | *NPTX1* | Neuronal pentraxin-1 | 46.3 | 11.9 |
| 2 | *MFAP4* | Microfibril-associated glycoprotein 4 | 28.1 | 6.6 |
| 3 | *CAMK2D* | Calcium/calmodulin-dependent protein kinase type II subunit delta | 11.7 | 3.5 |
| 4 | *HMGA2* | High mobility group protein HMGI-C | 6.8 | 4.2 |
| 5 | *KCNMA1* | Calcium-activated potassium channel subunit alpha-1 | 6.5 | 22.9 |
| 6 | *ADAMTS5* | A disintegrin and metalloproteinase with thrombospondin motifs 5 | 3.7 | 45.5 |
| 7 | *IL6R* | Interleukin-6 receptor subunit alpha | 3.4 | 4.0 |
|  | Downregulated genes | |  |  |
| 1 | *HOXA3* | Homeobox protein Hox-A3 | 0.001 | 0.016 |
| 2 | *PDPN* | Podoplanin | 0.001 | 0.051 |
| 3 | *PRKCZ* | Protein kinase C zeta type | 0.001 | 0.007 |
| 4 | *SKAP2* | Src kinase-associated phosphoprotein 2 | 0.001 | 0.026 |
| 5 | *FAM198B* | Golgi-associated kinase 1B | 0.001 | 0.017 |
| 6 | *CD200* | OX-2 membrane glycoprotein | 0.002 | 0.080 |
| 7 | *HOXB9* | Homeobox protein Hox-B9 | 0.002 | 0.088 |
| 8 | *QPRT* | Nicotinate-nucleotide pyrophosphorylase | 0.003 | 0.023 |
| 9 | *ABCC6* | Multidrug resistance-associated protein 6 | 0.003 | 0.042 |
| 10 | *CD40* | Tumor necrosis factor receptor superfamily member 5 | 0.003 | 0.053 |
| 11 | *CACNG6* | Voltage-dependent calcium channel gamma-6 subunit | 0.003 | 0.014 |
| 12 | *SLC18B1* | MFS-type transporter SLC18B1 | 0.004 | 0.028 |
| 13 | *HOXA4* | Homeobox protein Hox-A4 | 0.006 | 0.017 |
| 14 | *ALDH1A2* | Retinal dehydrogenase 2 | 0.006 | 0.003 |
| 15 | *PIANP* | PILR alpha-associated neural protein | 0.006 | 0.019 |
| 16 | *CLDN11* | Claudin-11 | 0.008 | 0.082 |
| 17 | *TRPV2* | Transient receptor potential cation channel subfamily V member 2 | 0.008 | 0.073 |
| 18 | *HOXA2* | Homeobox protein Hox-A2 | 0.009 | 0.036 |
| 19 | *IFNLR1* | Interferon lambda receptor 1 | 0.009 | 0.040 |
| 20 | *EPHX2* | Bifunctional epoxide hydrolase 2 | 0.010 | 0.034 |
| 21 | *CA11* | Carbonic anhydrase-related protein 11 | 0.010 | 0.035 |
| 22 | *RNF43* | E3 ubiquitin-protein ligase RNF43 | 0.010 | 0.004 |
| 23 | *C15orf48* | Normal mucosa of esophagus-specific gene 1 protein | 0.011 | 0.066 |
| 24 | *HOXA5* | Homeobox protein Hox-A5 | 0.012 | 0.006 |
| 25 | *SHROOM3* | Protein Shroom3 | 0.012 | 0.069 |
| 26 | *RNF182* | E3 ubiquitin-protein ligase RNF182 | 0.013 | 0.078 |
| 27 | *HMSD* | Serpin-like protein HMSD | 0.013 | 0.051 |
| 28 | *IQCA1* | Dynein regulatory complex protein 11 | 0.013 | 0.013 |
| 29 | *MATN3* | Matrilin-3 | 0.013 | 0.065 |
| 30 | *JAK3* | Tyrosine-protein kinase JAK3 | 0.013 | 0.021 |
| 31 | *ARSI* | Arylsulfatase I | 0.015 | 0.039 |
| 32 | *OBSL1* | Obscurin-like protein 1 | 0.015 | 0.045 |
| 33 | *DOCK8* | Dedicator of cytokinesis protein 8 | 0.015 | 0.018 |
| 34 | *KIF5A* | Kinesin heavy chain isoform 5A | 0.015 | 0.045 |
| 35 | *DNAJC12* | DnaJ homolog subfamily C member 12 | 0.016 | 0.028 |
| 36 | *VCAM1* | Vascular cell adhesion protein 1 | 0.016 | 0.004 |
| 37 | *FAM169A* | Soluble lamin-associated protein of 75 kDa | 0.017 | 0.072 |
| 38 | *TDRKH* | Tudor and KH domain-containing protein | 0.018 | 0.090 |
| 39 | *CFI* | Complement factor I | 0.018 | 0.003 |
| 40 | *FBXO27* | F-box only protein 27 | 0.019 | 0.089 |
| 41 | *PRKCQ-AS1* | PRKCQ antisense RNA 1 | 0.019 | 0.076 |
| 42 | *C1orf106* | Innate immunity activator protein | 0.020 | 0.075 |
| 43 | *BTBD11* | Ankyrin repeat and BTB/POZ domain-containing protein BTBD11 | 0.021 | 0.007 |
| 44 | *TMOD2* | Tropomodulin-2 | 0.021 | 0.081 |
| 45 | *HOTAIRM1* | HOXA transcript antisense RNA, myeloid-specific 1 | 0.021 | 0.028 |
| 46 | *DSC3* | Desmocollin-2 | 0.021 | 0.010 |
| 47 | *FAM149A* | Protein FAM149A | 0.021 | 0.046 |
| 48 | *KCTD14* | BTB/POZ domain-containing protein KCTD14 | 0.022 | 0.062 |
| 49 | *HOXC9* | Homeobox protein Hox-C9 | 0.023 | 0.036 |
| 50 | *ITGB3* | Integrin beta-3 | 0.023 | 0.042 |
| 51 | *HOXA-AS2* | HOXA cluster antisense RNA 2 | 0.024 | 0.020 |
| 52 | *ANKEF1* | Ankyrin repeat and EF-hand domain-containing protein 1 | 0.024 | 0.082 |
| 53 | *RARRES1* | Ankyrin repeat and EF-hand domain-containing protein 1 | 0.024 | 0.032 |
| 54 | *MANSC1* | MANSC domain-containing protein 1 | 0.025 | 0.067 |
| 55 | *VANGL2* | Vang-like protein 2 | 0.027 | 0.091 |
| 56 | *DCN* | Decorin | 0.028 | 0.054 |
| 57 | *PRCP* | Homo sapiens prolylcarboxypeptidase | 0.029 | 0.068 |
| 58 | *C7orf69* | Uncharacterized protein C7orf69 | 0.029 | 0.026 |
| 59 | *PCLO* | Protein piccolo | 0.029 | 0.090 |
| 60 | *AREG* | Amphiregulin | 0.031 | 0.044 |
| 61 | *STYK1* | Tyrosine-protein kinase STYK1 | 0.031 | 0.031 |
| 62 | *MMP24* | Matrix metalloproteinase-24 | 0.032 | 0.081 |
| 63 | *AMIGO2* | Amphoterin-induced protein 2 | 0.033 | 0.041 |
| 64 | *NFE2* | Transcription factor NF-E2 45 kDa subunit | 0.035 | 0.081 |
| 65 | *STK33* | Serine/threonine-protein kinase 33 | 0.037 | 0.067 |
| 66 | *RASIP1* | Ras-interacting protein 1 | 0.037 | 0.053 |
| 67 | *C19orf81* | Putative uncharacterized protein C19orf81 | 0.039 | 0.012 |
| 68 | *ATL1* | Atlastin-1 | 0.040 | 0.057 |
| 69 | *FAM221A* | Protein FAM221A | 0.043 | 0.084 |
| 70 | *TLR3* | Toll-like receptor 3 | 0.045 | 0.090 |
| 71 | *MGARP* | Protein MGARP | 0.046 | 0.047 |
| 72 | *SLC40A1* | Solute carrier family 40 member 1 | 0.046 | 0.013 |
| 73 | *IFT27* | Intraflagellar transport protein 27 homolog | 0.047 | 0.052 |
| 74 | *ACSF2* | Acyl-CoA synthetase family member 2, mitochondrial | 0.048 | 0.083 |
| 75 | *SMPDL3B* | Acid sphingomyelinase-like phosphodiesterase 3b | 0.049 | 0.066 |
| 76 | *SNAP25* | Synaptosomal-associated protein 25 | 0.049 | 0.038 |
| 77 | *WDR86* | WD repeat-containing protein 86 | 0.050 | 0.024 |
| 78 | *CLEC11A* | C-type lectin domain family 11 member A | 0.050 | 0.085 |
| 79 | *RCN3* | Reticulocalbin-3 | 0.051 | 0.071 |
| 80 | *SLC4A3* | Anion exchange protein 3 | 0.052 | 0.051 |
| 81 | *SEZ6L2* | Seizure 6-like protein 2 | 0.054 | 0.006 |
| 82 | *CYP2S1* | Cytochrome P450 2S1 | 0.055 | 0.022 |
| 83 | *MAGEH1* | Melanoma-associated antigen H1 | 0.063 | 0.061 |
| 84 | *SH3BGRL2* | SH3 domain-binding glutamic acid-rich-like protein 2 | 0.066 | 0.070 |
| 85 | *F11R* | Junctional adhesion molecule A | 0.067 | 0.075 |
| 86 | *ASPHD1* | Aspartate beta-hydroxylase domain-containing protein 1 | 0.071 | 0.064 |
| 87 | *SFRP1* | Secreted frizzled-related protein 1 | 0.073 | 0.043 |
| 88 | *SULF2* | Extracellular sulfatase Sulf-2 | 0.073 | 0.042 |
| 89 | *JPH4* | Junctophilin-4 | 0.081 | 0.032 |
| 90 | *GCA* | Grancalcin | 0.083 | 0.064 |
| 91 | *ADAMTS10* | A disintegrin and metalloproteinase with thrombospondin motifs 10 | 0.091 | 0.090 |
