# Supplementary material for: CAMK2D: a novel molecular target for BAP1-deficient malignant mesothelioma
Source: Cell Death Discov. 2023 Jul 21;9:257. doi: 10.1038/s41420-023-01552-5 (PMC10362017; doi:10.1038/s41420-023-01552-5)
Supplement: Supplementary file 3 — Table S2. Summary of immunohistochemistry in this study [file 41420_2023_1552_MOESM3_ESM.docx]

Table S2. Summary of immunohistochemistry in this study

| Cases No. | Age | Sex | Organ  (Anatomic Site) | Pathology  diagnosis | BAP1 | CAMK2D |  |
| --- | --- | --- | --- | --- | --- | --- | --- |
| 1 | M | 34 | Pleura | Thoracic cavity | 2+ | 0 |  |
| 2 | M | 67 | Pleura | Thoracic cavity | 3+ | 0 |  |
| 3 | F | 51 | Blood vessel | Pulmonary artery | 3+ | 0 |  |
| 4 | F | 70 | Abdominal cavity | Epithelial malignant | 1+ | 3+ |  |
| 5 | M | 60 | Abdominal cavity | Abdominal membrane | 1+ | 3+ |  |
| 6 | M | 5 | Abdominal cavity | Malignant mesothelioma | 0 | 3+ |  |
| 7 | M | 60 | Abdominal cavity | abdominal membrane | 2+ | 0 |  |
| 8 | F | 47 | Abdominal cavity | Malignant mesothelioma | 2+ | 0 |  |
| 9 | M | 33 | Abdominal cavity | Abdominal membrane | 0 | 3+ |  |
| 10 | F | 38 | Abdominal cavity | Malignant mesothelioma | 0 | 1+ |  |
| 11 | F | 71 | Mesentery | Malignant mesothelioma | 0 | 1+ |  |
| 12 | F | 29 | Omentum | Malignant mesothelioma | 0 | 2+ |  |
| 13 | M | 63 | Retroperitoneum | Malignant mesothelioma | 0 | 2+ |  |
| 14 | M | 48 | Abdominal cavity | Abdominal membrane | 0 | 3+ |  |
| 15 | M | 43 | Pericardium | Epithelial malignant | 0 | 2+ |  |
| 16 | F | 32 | Abdominal cavity | Abdominal cavity | 2+ | 2+ |  |
| 17 | F | 48 | Omentum | Malignant mesothelioma | 0 | 2+ |  |
| 18 | F | 23 | Lung | Malignant mesothelioma | 0 | 2+ |  |
| 19 | F | 18 | Lung | Malignant mesothelioma | 0 | 1+ |  |
| 20 | F | 56 | Pleura | Malignant mesothelioma | 2+ | 2+ |  |
| 21 | F | 58 | Pleura | Malignant mesothelioma | 0 | 2+ |  |
| 22 | F | 22 | Pleura | Malignant mesothelioma | 2+ | 2+ |  |
| 23 | F | 70 | Pleura | Malignant mesothelioma | 0 | 2+ |  |
| 24 | M | 47 | Pleura | Malignant mesothelioma | 0 | 3+ |  |
| 25 | M | 49 | Pleura | Malignant mesothelioma | 0 | 3+ |  |
| 26 | F | 64 | Pleura | Epithelial malignant | 0 | 0 |  |
| 27 | M | 49 | Pleura | Malignant mesothelioma | 1+ | 3+ |  |
| 28 | M | 83 | Pleura | Malignant mesothelioma | 0 | 2+ |  |
| 29 | M | 50 | Pericardium | Malignant mesothelioma | 0 | 3+ |  |
| 30 | M | 43 | Pericardium | Malignant mesothelioma | 0 | 2+ |  |
| 31 | 64 | M | Pleura | Malignant mesothelioma of chest wall | 1+ | 0 |  |
| 32 | 48 | M | Pleura | Malignant mesothelioma | 0 | 2+ |  |
| 33 | 58 | F | Pleura | Malignant mesothelioma | 0 | 2+ |  |
| 34 | 49 | F | Pleura | Malignant mesothelioma | 1+ | 0 |  |
| 35 | 22 | M | Pleura | Malignant mesothelioma | 0 | 2+ |  |
| 36 | 54 | F | Mediastinum | Malignant mesothelioma | 0 | 0 |  |
| 37 | 49 | M | Pleura | Malignant mesothelioma | 0 | 2+ |  |
| 38 | 32 | M | Pleura | Malignant mesothelioma | 1+ | 0 |  |
| 39 | 29 | M | Pleura | Malignant mesothelioma with necrosis | 0 | 2+ |  |
| 40 | 53 | F | Thoracic cavity | Malignant mesothelioma | 0 | 1+ |  |
| 41 | 31 | F | Thoracic cavity | Malignant mesothelioma | 0 | 2+ |  |
| 42 | 70 | F | Pleura | Malignant mesothelioma | 0 | 2+ |  |
| 43 | 47 | M | Pleura | Malignant mesothelioma | 0 | 1+ |  |
| 44 | 60 | M | Pleura | Malignant mesothelioma | 1+ | 3+ |  |
| 45 | 46 | M | Thoracic cavity | Malignant mesothelioma | 0 | 2+ |  |
| 46 | 35 | M | Pleura | Malignant mesothelioma | 0 | 2+ |  |
| 47 | 83 | M | Pleura | Malignant mesothelioma | 0 | 2+ |  |
| 48 | 56 | M | Mediastinum | Malignant mesothelioma of mediastinal pleura | 1+ | 0 |  |
| 49 | 67 | F | Thoracic cavity | Malignant mesothelioma | 0 | 2+ |  |
| 50 | 40 | F | Heart | Malignant mesothelioma of left cardiac atrium | 0 | 1+ |  |
| 51 | 50 | F | Heart | Malignant mesothelioma of left cardiac atrium | 0 | 2+ |  |
| 52 | 43 | F | Heart | Malignant mesothelioma of pericardium | 0 | 2+ |  |
| 53 | 43 | F | Heart | Malignant mesothelioma of pericardium | 0 | 2+ |  |
| 54 | 50 | F | Heart | Malignant mesothelioma of pericardium | 0 | 2+ |  |
| 55 | 57 | M | Peritoneum | Malignant mesothelioma | 0 | 3+ |  |
| 56 | 29 | M | Peritoneum | Malignant mesothelioma | 0 | 2+ |  |
| 57 | 71 | M | Mesentery | Malignant mesothelioma of mesostenium | 0 | 2+ |  |
| 58 | 63 | F | Peritoneum | Malignant mesothelioma | 1+ | 0 |  |
| 59 | 48 | F | Peritoneum | Malignant mesothelioma | 1+ | 0 |  |
| 60 | 60 | F | Peritoneum | Malignant mesothelioma | 0 | 2+ |  |
| 61 | 60 | F | Peritoneum | Malignant mesothelioma | 0 | 2+ |  |
| 62 | 47 | M | Peritoneum | Malignant mesothelioma | 0 | 3+ |  |
| 63 | 33 | F | Peritoneum | Malignant mesothelioma | 0 | 0 |  |
| 64 | 47 | M | Colon | Malignant mesothelioma of mesocolon | 0 | 2+ |  |
| 65 | 44 | F | Colon | Malignant mesothelioma of mesocolon | 0 | 1+ |  |
| 66 | 53 | M | Greater omentum | Malignant mesothelioma with necrosis | 1+ | 0 |  |
| 67 | 41 | M | Greater omentum | Malignant mesothelioma | 0 | 1+ |  |
| 68 | 60 | M | Greater omentum | Malignant mesothelioma | 0 | 1+ |  |
| 69 | 60 | M | Greater omentum | Malignant mesothelioma | 0 | 1+ |  |
| 70 | 76 | F | Pelvic cavity | Malignant mesothelioma of hypogastrium | 0 | 2+ |  |
| 71 | 78 | M | Pelvic cavity | Malignant mesothelioma with necrosis of hypogastrium | 0 | 2+ |  |
| 72 | 41 | F | Pelvic cavity | Malignant mesothelioma of hypogastrium | 0 | 2+ |  |
| 73 | 28 | M | Peritoneum | Malignant mesothelioma of retroperitoneum | 0 | 1+ |  |
| 74 | 5 | F | Abdominal cavity | Malignant mesothelioma of epigastrium | 0 | 2+ |  |
| 75 | 56 | F | Colon | Malignant mesothelioma of mesocolon | 1+ | 0 |  |
| 76 | 45 | M | Peritoneum | Malignant mesothelioma | 0 | 2+ |  |
| 77 | 69 | M | Peritoneum | Malignant mesothelioma | 0 | 1+ |  |
| 78 | 78 | M | Pelvic cavity | Malignant mesothelioma of hypogastrium | 1+ | 0 |  |
| 79 | 33 | M | Peritoneum | Malignant mesothelioma of retroperitoneum | 0 | 1+ |  |
| 80 | 5 | F | Abdominal cavity | Malignant mesothelioma | 0 | 1+ |  |

The intensity of the positive signal for BAP1 and CAMK2D were evaluated by two investigators.
