# Supplementary material for: CAMK2D: a novel molecular target for BAP1-deficient malignant mesothelioma
Source: Cell Death Discov. 2023 Jul 21;9:257. doi: 10.1038/s41420-023-01552-5 (PMC10362017; doi:10.1038/s41420-023-01552-5)
Supplement: Supplementary file 4 — Table S3. Effect of 363 compounds on the proliferation of BAP1-KO and Parental Met-5A cells [file 41420_2023_1552_MOESM4_ESM.docx]

Table S3. Effect of 363 compounds on the proliferation of *BAP1*-KO and Parental Met-5A cells.

| No | Category | Compound | MeT-5A (Parent) (% cells) | *BAP1*-KO | Relative cell survival |
| --- | --- | --- | --- | --- | --- |
|  |  |  |  | (% cells) | *BAP1*-KO-parent (%cells) |
| 1 | Blank (control) | DMSO DMSO | 83.88 | 92.82 | 8.94 |
| 2 | antitumor | 5-FU | 67.78 | 84.34 | 16.56 |
| 3 | antitumor | Bestatin | 77.45 | 88.5 | 11.05 |
| 4 | antitumor (DNA) | Bleomycin sulfate | 22.88 | 36.89 | 14.01 |
| 5 | antitumor (DNA) | Cisplatin | 84.6 | 93.05 | 8.45 |
| 6 | antitumor (DHFR) | Methotrexate | 41.39 | 59.81 | 18.42 |
| 7 | antitumor (DNA) | Mitomycin C | 14.35 | 18.52 | 4.17 |
| 8 | antitumor (tubulin) | Vinblastine sulfate | 31.9 | 54.04 | 22.14 |
| 9 | antitumor (tubulin) | Paclitaxel | 68.69 | 77.48 | 8.79 |
| 10 | antitumor (AR) | Flutamide | 81.14 | 96.18 | 15.04 |
| 11 | antitumor (DNA) | Daunorubicin, HCl | 15.47 | 22.9 | 7.43 |
| 12 | antitumor (DNA) | Doxorubicin, HCl | 22.11 | 26.25 | 4.14 |
| 13 | antitumor (ER) | Tamoxifen, citrate | 37.94 | 39.97 | 2.03 |
| 14 | antitumor (RNA) | Actinomycin D | 20.73 | 29.03 | 8.3 |
| 15 | antitumor (topo I) | Camptothecin | 28.45 | 46.48 | 18.03 |
| 16 | antitumor (topo I/II) | Aclarubicin | 19.01 | 27.87 | 8.86 |
| 17 | antitumor (topo II) | Etoposide (VP-16) | 50.27 | 73.61 | 23.34 |
| 18 | actin filament | Cytochalasin D | 88.95 | 93.01 | 4.06 |
| 19 | adenylcyclase | 2',5'-dideoxyadenosine | 90.95 | 99.55 | 8.6 |
| 20 | AKT | AKT inhibitor | 85.29 | 91.5 | 6.21 |
| 21 | AKT | NL-71-101 | 92.79 | 93.14 | 0.35 |
| 22 | Bcr-Abl | AG957 | 70.76 | 90.28 | 19.52 |
| 23 | caspase | Z-VAD-FMK | 77.32 | 88.67 | 11.35 |
| 24 | CDC2 | Kenpaullone | 55.37 | 69.7 | 14.33 |
| 25 | CDK2 | Purvalanol A | 48.55 | 60.22 | 11.67 |
| 26 | CDK4 | 3-ATA | 60.7 | 84.12 | 23.42 |
| 27 | CDKs | Olomoucine | 81.31 | 87.98 | 6.67 |
| 28 | CKII | TBB | 85.41 | 90.51 | 5.1 |
| 29 | COX-1 | Sulindac sulfide | 85.16 | 89.08 | 3.92 |
| 30 | COX-1 | Valeryl salicylate | 83.25 | 93.09 | 9.84 |
| 31 | COX-2 | NS-398 | 81.09 | 92.16 | 11.07 |
| 32 | COX | Sodium salicylate | 87.96 | 95.27 | 7.31 |
| 33 | cyclicphosphodiesterase | Theophylline | 83.93 | 92.83 | 8.9 |
| 34 | DNA methyltransferase | Azacytidine | 82.53 | 94.27 | 11.74 |
|  |  |  |  |  |  |
| 35 | DNA polymerase | Aphidicolin | 45.28 | 66.03 | 20.75 |
| 36 | EGFR | AG1478 | 84.04 | 86.5 | 2.46 |
| 37 | EGFR, topoII | Genistein | 86.32 | 89.47 | 3.15 |
| 38 | farnesyltransferase | Manumycin A | 32.21 | 79.73 | 47.52 |
| 39 | farnesyltransferase | FTI-276 | 77.9 | 87.57 | 9.67 |
| 40 | Flk-1 | SU1498 | 68.26 | 64.48 | -3.78 |
| 41 | geranylgeranyltransferase I | GGTI-286 | 64.35 | 71.6 | 7.25 |
| 42 | GR | Dexamethasone | 83.75 | 91.85 | 8.1 |
| 43 | GSK-3 | GSK-3 inhibitor II | 77.37 | 90.34 | 12.97 |
| 44 | HDAC | Scriptaid | 23.81 | 37.61 | 13.8 |
| 45 | HDAC | Trichostatin A | 24.19 | 36.49 | 12.3 |
| 46 | HER2 (erbB2/neu), EGFR | AG825 | 82.95 | 92.33 | 9.38 |
| 47 | protein synthesis | Cycloheximide | 34.5 | 49.48 | 14.98 |
| 48 | HMG-CoA reductase | Lovastatin | 39.76 | 55.5 | 15.74 |
| 49 | HSP90 | Radicicol | 25.72 | 43.65 | 17.93 |
| 50 | HSP90 | 17-AAG | 32.49 | 57.37 | 24.88 |
| 51 | IGF-1R | AG1024 | 88.42 | 90.2 | 1.78 |
| 52 | iNOS | 1400W, HCl | 77.91 | 85.48 | 7.57 |
| 53 | iNOS | AMT, HCl | 88.87 | 92.1 | 3.23 |
| 54 | Jak-2 | AG490 | 92.97 | 92.97 | 0 |
| 55 | Jak-2 | Cucurbitacin I | 22.65 | 36.14 | 13.49 |
| 56 | JNK | SP600125 | 71.98 | 80.56 | 8.58 |
| 57 | lck (p56), TYK | Damnacanthal | 46.99 | 80.37 | 33.38 |
| 58 | MEK | PD 98059 | 82.41 | 88.01 | 5.6 |
| 59 | MEK | U0126 | 87.63 | 83.64 | -3.99 |
| 60 | methionine aminopeptidase | Fumagillin | 68.08 | 83.04 | 14.96 |
| 61 | MMP | GM 6001 | 84.19 | 87.92 | 3.73 |
| 62 | NF-kB | N-Acetyl-L-cysteine | 84.49 | 93.59 | 9.1 |
| 63 | NOS | Aminoguanidine, HCl | 85.15 | 91.15 | 6 |
| 64 | NOS | L-NMMA | 86.58 | 93.32 | 6.74 |
| 65 | p38 (MAPK) | PD169316 | 80.42 | 86.33 | 5.91 |
| 66 | p38 (MAPK) | SB 203580 | 75.8 | 91.69 | 15.89 |
| 67 | p70 S6K | Rapamycin | 74.45 | 77.37 | 2.92 |
| 68 | PARP | NU1025 | 82.48 | 89.81 | 7.33 |
| 69 | PARP-1 | Benzamide | 83.88 | 91.36 | 7.48 |
| 70 | PC-PLC | D609 | 85.56 | 91.67 | 6.11 |
| 71 | PDE | IBMX | 83.19 | 90.53 | 7.34 |
|  |  |  |  |  |  |
| 72 | PDE (cAMP) | Ro-20-1724 | 87.14 | 93.2 | 6.06 |
| 73 | PDE (cGMP) | Zaprinast | 84.83 | 89.18 | 4.35 |
| 74 | PDGFR | AG1296 | 63.99 | 88.29 | 24.3 |
| 75 | PI3K | LY294002 | 54.6 | 63.04 | 8.44 |
| 76 | PI3K | Wortmannin | 71.82 | 81.9 | 10.08 |
| 77 | PKA | H-89, HCl | 53.55 | 75.32 | 21.77 |
| 78 | PKC | Bisindolymaleimide I, HCl | 22.65 | 47.52 | 24.87 |
| 79 | PKC, PKA | H-7 | 73.07 | 86.35 | 13.28 |
| 80 | PKC, PKA, PKG, MLCK | Staurosporine | 13.72 | 19.96 | 6.24 |
| 81 | PLA2 | cPLA2inhibitor | 89.03 | 90.76 | 1.73 |
| 82 | PLA2 | OBAA | 61.93 | 70.17 | 8.24 |
| 83 | PP2A | Cantharidin | 84.98 | 89.47 | 4.49 |
| 84 | PP2A | Cytostatin | 81.97 | 86.87 | 4.9 |
| 85 | PP2B/cyclophilin | Cyclosporin A | 94.14 | 82.03 | -12.11 |
| 86 | PP2B/FKBP | FK-506 | 81.08 | 91.65 | 10.57 |
| 87 | proteasome | MG-132 | 25.6 | 41.38 | 15.78 |
| 88 | proteasome | Lactacystin | 77.62 | 92.74 | 15.12 |
| 89 | ribonucleotide reductase | Hydroxyurea | 88 | 94.29 | 6.29 |
| 90 | ROCK | HA1077 | 76.33 | 85.17 | 8.84 |
| 91 | ROCK | Y27632 | 77.73 | 83.72 | 5.99 |
| 92 | Src, Fyn, Lck | PP1 (analog) | 86.13 | 90.74 | 4.61 |
| 93 | Src, Fyn, Lck | PP-H | 91.84 | 90.82 | -1.02 |
| 94 | tubulin depolymerization | Nocodazole | 37.61 | 63.59 | 25.98 |
| 95 | tyr phosphatase (PTP) | Dephostatin | 89.66 | 102.47 | 12.81 |
| 96 | p53 | Pifithrin-a (cyclic) | 87.73 | 70.81 | -16.92 |
| 97 | p53 activator | PRIMA-1 | 86.81 | 93.88 | 7.07 |
| 98 | 5α-reductase | Finasteride | 89.4 | 94.25 | 4.85 |
| 99 | aromatase | Aminoglutethimide | 87.32 | 100.36 | 13.04 |
| 100 | aromatase | Formestane | 86.74 | 94.92 | 8.18 |
| 101 | progesterone receptor | Mifepristone | 86.97 | 98.7 | 11.73 |
| 102 | acetyl-CoA carboxylase | TOFA | 86.73 | 98.49 | 11.76 |
| 103 | aminopeptidase A | Amastatin | 86.86 | 97.93 | 11.07 |
| 104 | aminopeptidase M | Actinonin | 85.36 | 94.69 | 9.33 |
| 105 | F1-ATPase | Oligomycin | 24.2 | 14.83 | -9.37 |
| 106 | V-ATPase | Bafilomycin A1 | 13.72 | 14.63 | 0.91 |
| 107 | Bcl-2 | HA 14-1 | 87.19 | 96.28 | 9.09 |
| 108 | Bcl-XL | BH3I-1 | 89.22 | 97.38 | 8.16 |
|  |  |  |  |  |  |
| 109 | Burton's tyrosine kinase(BTK) | LFM-A13 | 89.58 | 97.21 | 7.63 |
| 110 | Burton's tyrosine kinase(BTK) | Terreic acid | 81.26 | 98.47 | 17.21 |
| 111 | calpain | E-64d | 88.31 | 97.77 | 9.46 |
| 112 | calpain, cathepsin B, L | ALLN | 96.99 | 101.85 | 4.86 |
| 113 | cathepsin B | CA-074 | 90.43 | 103.69 | 13.26 |
| 114 | cathepsin D | Pepstatin A | 92.91 | 103.96 | 11.05 |
| 115 | cathepsin G | Z-GLF-CMK | 25.88 | 41.35 | 15.47 |
| 116 | CCR2 | RS 102895 | 87.42 | 95.31 | 7.89 |
| 117 | CCR3 | SB 328437 | 91.23 | 97.87 | 6.64 |
| 118 | CXCR2 | SB 225002 | 51.98 | 67.38 | 15.4 |
| 119 | CXCR4 | AMD3100 octahydrochloride | 84.59 | 96.11 | 11.52 |
| 120 | Cdc25 | NSC95397 | 29.72 | 115.37 | 85.65 |
| 121 | Cdc25A | SC-αασ9 | 98.79 | 106.95 | 8.16 |
| 122 | Na channel | Amiloride | 92.16 | 106.13 | 13.97 |
| 123 | Na channel | Lidocaine | 94.54 | 102.69 | 8.15 |
| 124 | Na ionophore | Monensin | 35.85 | 53 | 17.15 |
| 125 | Na/K ATPase | Ouabain | 30.43 | 41.13 | 10.7 |
| 126 | Na/K/Mg ATPase | Sanguinarine | 13.28 | 19.96 | 6.68 |
| 127 | K channel | Glibenclamide | 86.76 | 97.69 | 10.93 |
| 128 | K channel | Dequalinium | 17.37 | 21.35 | 3.98 |
| 129 | K channel opener | Diazoxide | 92.66 | 99.92 | 7.26 |
| 130 | K ionophore | Valinomycin | 16.29 | 17.3 | 1.01 |
| 131 | K ionophore | Nigericin | 44.98 | 45.89 | 0.91 |
| 132 | Ca channel | Diltiazem | 91.49 | 101.45 | 9.96 |
| 133 | Ca channel | Nifedipine | 90.67 | 97.17 | 6.5 |
| 134 | Ca channel, MDR | Verapamil | 85.26 | 90.92 | 5.66 |
| 135 | MDR | PGP-4008 | 50.66 | 56.21 | 5.55 |
| 136 | BCRP | Fumitremorgin C | 94.16 | 96.43 | 2.27 |
| 137 | Ca ionophore | A23187 | 13.91 | 18.5 | 4.59 |
| 138 | Ca ionophore | Ionomycin | 17.6 | 28.59 | 10.99 |
| 139 | Ca-ATPase | Thapsigargin | 21.58 | 25.9 | 4.32 |
| 140 | Ca-ATPase | t-Butylhydroquinone (BHQ) | 111.61 | 121.56 | 9.95 |
| 141 | Cl channel | N-phenylanthranilic acid | 88.11 | 98.27 | 10.16 |
| 142 | Cl channel | DIDS | 89.05 | 96.47 | 7.42 |
| 143 | Chk 1 | SB 218078 | 33.28 | 48.66 | 15.38 |
| 144 | Chk 1, 2 | Debromohymenialdisine (DBH) | 95.44 | 96.2 | 0.76 |
|  |  |  |  |  |  |
| 145 | mitochondrial complex I | Rotenone | 43.96 | 57.2 | 13.24 |
| 146 | mitochondrial complex III | Antimycin A1 | 59.97 | 58.61 | -1.36 |
| 147 | CRM1 | Leptomycin B* | 33.58 | 52.44 | 18.86 |
| 148 | DAG kinase | R59022 | 97.44 | 92 | -5.44 |
| 149 | DAG kinase | Dioctanoylglycol | 92.33 | 99.4 | 7.07 |
| 150 | DAG lipase | RHC80267 | 90.58 | 91.73 | 1.15 |
| 151 | DAG acyltransferase (DGAT) | Xanthohumol | 16.18 | 19.88 | 3.7 |
| 152 | fatty acid synthase (FAS) | C75 | 88.8 | 93.67 | 4.87 |
| 153 | FAS | Cerulenin | 80.83 | 84.99 | 4.16 |
| 154 | glycosylation | Tunicamycin | 35.88 | 53.08 | 17.2 |
| 155 | glucosidase I, II | Deoxynojirimycin | 92.97 | 102.73 | 9.76 |
| 156 | a-mannosidase | Swainsonine | 93.91 | 103.56 | 9.65 |
| 157 | guanylate cyclase | LY 83583 | 27.7 | 44.66 | 16.96 |
| 158 | guanylate cyclase | ODQ | 92.94 | 98.37 | 5.43 |
| 159 | HAT | Anacardic acid | 96.63 | 105.4 | 8.77 |
| 160 | HIF | Chetomin | 13.64 | 15.78 | 2.14 |
| 161 | HIF-1a hydroxylase | Dimethyloxalylglycine | 95.11 | 100.75 | 5.64 |
| 162 | kinesin Eg5 | HR22C16 | 31.11 | 51.8 | 20.69 |
| 163 | kinesin Eg5 | Monastrol | 94.39 | 102.28 | 7.89 |
| 164 | lipoxygenase | Nordihydroguaiaretic acid (NDGA) | 93.32 | 102.88 | 9.56 |
| 165 | 12, 15-lipoxygenase | ETYA | 92.12 | 100.21 | 8.09 |
| 166 | 12-lipoxygenase | Baicalein | 63.86 | 83.56 | 19.7 |
| 167 | Mdm2 | Nutlin-3 | 96.99 | 97.23 | 0.24 |
| 168 | Mdm2 | MDM2 inhibitor | 99.56 | 100.6 | 1.04 |
| 169 | monoamine oxidase | Phenelzine | 91.64 | 100.58 | 8.94 |
| 170 | monoamine oxdase B | Deprenyl | 93.48 | 97.27 | 3.79 |
| 171 | mitochondrial permeability transition pore (MPTP) | Decylubiquinone | 89.55 | 102.76 | 13.21 |
| 172 | MPTP | Ro 5-4864 | 86.69 | 94.44 | 7.75 |
| 173 | MPTP opener | Lonidamine | 92.26 | 96.09 | 3.83 |
| 174 | myosin light chain kinase | ML-7 | 79.86 | 88.29 | 8.43 |
| 175 | O6-methylguanine-DNA methyltransferase (MGMT) | Benzylguanine | 88.14 | 93.32 | 5.18 |
| 176 | ornithine decarboxylase (ODC) | DFMO | 92.53 | 97.38 | 4.85 |
| 177 | PKG | KT 5823 | 84.31 | 84.82 | 0.51 |
| 178 | PKG | Rp-8-CPT-cGMPS | 78.67 | 86.66 | 7.99 |
| 179 | PPAR-a | MK 886 | 92.33 | 96.01 | 3.68 |
|  |  |  |  |  |  |
| 180 | PPAR-a activator | Clofibrate | 91.72 | 99.59 | 7.87 |
| 181 | PPAR-g | BADGE | 91.65 | 95.39 | 3.74 |
| 182 | PPAR-g activator | Troglitazone | 98.44 | 94.36 | -4.08 |
| 183 | reverse transcriptase | AZT | 87.72 | 95.87 | 8.15 |
| 184 | reverse transcriptase | Nalidixic acid | 90.65 | 93.67 | 3.02 |
| 185 | RNA polymerase | a-Amanitin | 38.68 | 56.17 | 17.49 |
| 186 | telomerase | MST-312 | 46.36 | 63.72 | 17.36 |
| 187 | telomerase | b-Rubromycin | 70.35 | 88 | 17.65 |
| 188 | TGF-b receptor | SB 431542 | 91.74 | 99.07 | 7.33 |
| 189 | spermidine/spermine N1-acetyltransferase (SSAT) activator | N1,N12-Diethylspermine (BESpm) | 85 | 87.63 | 2.63 |
| 190 | sphingosine N-acyltransferase | Fumonisin B1 | 83.55 | 91.94 | 8.39 |
| 191 | AK | ABT-702 | 63.38 | 101.06 | 37.68 |
| 192 | AKT | Akt Inhibitor IV | 10.64 | 13.96 | 3.32 |
| 193 | AKT | Akt Inhibitor VIII, Isozyme-Selective, Akti-1/2 | 41.49 | 69.92 | 28.43 |
| 194 | AKT | Akt Inhibitor XI | 38.16 | 76.61 | 38.45 |
| 195 | AMPK | compound C | 31.62 | 52.88 | 21.26 |
| 196 | ATM | ATM/ATR kinase inhibitor | 12.04 | 21.41 | 9.37 |
| 197 | ATM | ATM kinase inhibitor | 71.5 | 70.21 | -1.29 |
| 198 | Aurora | Aurora kinase/cdk inhibitor | 47.02 | 59.13 | 12.11 |
| 199 | Aurora | Aurora kinase inhibitor II | 105.48 | 102.61 | -2.87 |
| 200 | Aurora | Aurora kinase inhibitor III | 44.22 | 53.7 | 9.48 |
| 201 | Bcr-abl | AG957 | 74.7 | 95.97 | 21.27 |
| 202 | BTK | LFM-A13 | 94.77 | 95.1 | 0.33 |
| 203 | BTK | Terreic acid | 98.24 | 98.93 | 0.69 |
| 204 | CAMKII | KN-93 | 82.43 | 29.02 | -53.41 |
| 205 | CAMKII | KN-62 | 92.86 | 89.52 | -3.34 |
| 206 | CAMKII | Lavendustin C | 104.8 | 103.73 | -1.07 |
| 207 | CDK | Kenpaullone | 70.81 | 90.2 | 19.39 |
| 208 | CDK | purvalanol A | 55.13 | 73.73 | 18.6 |
| 209 | CDK | Olomoucine | 94.42 | 93.76 | -0.66 |
| 210 | CDK | Alsterpaullone, 2-cyanoethyl | 38.45 | 46.34 | 7.89 |
| 211 | CDK | Cdk1/2 inhibitor III | 35.24 | 53.71 | 18.47 |
| 212 | CDK | Cdk2/9 inhibitor | 42.94 | 53.5 | 10.56 |
| 213 | CDK | NU6102 | 70.93 | 79.88 | 8.95 |
| 214 | CDK | Cdk4 inhibitor | 34.8 | 43.92 | 9.12 |
|  |  |  |  |  |  |
| 215 | CDK | NSC625987 | 97.12 | 97.29 | 0.17 |
| 216 | Chk | SB218078 | 35.98 | 50.09 | 14.11 |
| 217 | Chk | isogranulatimide | 101 | 90.05 | -10.95 |
| 218 | Chk | Chk2 inhibitor | 94.92 | 90.53 | -4.39 |
| 219 | Chk | Chk2 inhibitor II | 89.89 | 66.66 | -23.23 |
| 220 | CK | Ellagic acid | 92.71 | 90.01 | -2.7 |
| 221 | CK | TBB | 93.15 | 92.87 | -0.28 |
| 222 | CK | DMAT | 73.02 | 95.39 | 22.37 |
| 223 | CK | D4476 | 93.6 | 98.23 | 4.63 |
| 224 | Clk | TG003 | 91.82 | 94.34 | 2.52 |
| 225 | DGK | Diacylglycerol kinase inhibitor II | 101.57 | 98.31 | -3.26 |
| 226 | DNA-PK | IC60211 | 93.88 | 94.98 | 1.1 |
| 227 | eEF2 | TX-1918 | 57.34 | 90.84 | 33.5 |
| 228 | EGFR | BPIQ-Ⅱ | 90.65 | 84.82 | -5.83 |
| 229 | EGFR | AG1478 | 96.4 | 94.29 | -2.11 |
| 230 | EGFR | AG490 | 94.42 | 91.44 | -2.98 |
| 231 | FGFR | SU4984 | 90.88 | 92.95 | 2.07 |
| 232 | FGFR | SU5402 | 78.37 | 80.21 | 1.84 |
| 233 | Flt-3 | Flt-3 Inhibitor | 56.82 | 53.04 | -3.78 |
| 234 | Fms | cFMS Receptor Tyrosine Kinase Inhibitor | 95.1 | 92.39 | -2.71 |
| 235 | Fyn | SU6656 | 66.99 | 77.85 | 10.86 |
| 236 | GSK | GSK-3 inhibitor IX | 93.93 | 92.02 | -1.91 |
| 237 | GSK | 1-Azakenpaullone | 59.77 | 68.12 | 8.35 |
| 238 | GSK | indirubin-3'-monoxime | 59.77 | 71.33 | 11.56 |
| 239 | HER2 | AG825 | 102.58 | 106.89 | 4.31 |
| 240 | IGF-IR | AG1024 | 105.15 | 102.34 | -2.81 |
| 241 | IGF-IR | AGL 2263 | 106.7 | 98.49 | -8.21 |
| 242 | IKK | BMS-345541 | 37.63 | 50.48 | 12.85 |
| 243 | IKK | IKK-2 inhibitor VI | 42.36 | 59.42 | 17.06 |
| 244 | IRAK | IRAK-1/4 inhibitor | 95.26 | 97.63 | 2.37 |
| 245 | Jak | JAK Inhibitor I | 105.18 | 93.32 | -11.86 |
| 246 | Jak | JAK3 Inhibitor VI | 104.36 | 97.87 | -6.49 |
| 247 | JNK | SP600125 | 91.26 | 92.87 | 1.61 |
| 248 | JNK | JNK inhibitor VIII | 95.02 | 98.74 | 3.72 |
| 249 | Lck | Damnacanthal | 83.65 | 89.66 | 6.01 |
| 250 | Lck | PP2 | 66.73 | 80.72 | 13.99 |
| 251 | MAPK | ERK inhibitor II | 85.95 | 87.57 | 1.62 |
|  |  |  |  |  |  |
| 252 | MEK | PD98059 | 96.2 | 97.17 | 0.97 |
| 253 | MEK | U-0126 | 99.02 | 90.67 | -8.35 |
| 254 | MEK | MEK inhibitor I | 93.22 | 83.54 | -9.68 |
| 255 | Met | SU11274 | 108.06 | 96.16 | -11.9 |
| 256 | MLCK | ML-7 | 83.73 | 92.08 | 8.35 |
| 257 | p38 | SB202190 | 86.58 | 92.47 | 5.89 |
| 258 | p38 | SB239063 | 89.51 | 87.78 | -1.73 |
| 259 | PDGFR | AG1296 | 88.79 | 95.76 | 6.97 |
| 260 | PDGFR | SU11652 | 11.55 | 12.97 | 1.42 |
| 261 | PDGFR | PDGF receptor tyrosine kinase inhibitor V | 103.62 | 92.31 | -11.31 |
| 262 | PDGFR | PDGF receptor tyrosine kinase inhibitor IV | 51.9 | 76.36 | 24.46 |
| 263 | PI3K | LY-294002 | 105.91 | 103.15 | -2.76 |
| 264 | PI3K | Wortmannin | 103.19 | 94.91 | -8.28 |
| 265 | PKA | H-89 | 104.39 | 95.99 | -8.4 |
| 266 | PKA | 4-cyano-3-methylisoquinoline | 104.92 | 98.29 | -6.63 |
| 267 | PKC | Bisindolymaleimide I, HCl | 32.52 | 59.19 | 26.67 |
| 268 | PKC | Go7874 | 11.4 | 13.57 | 2.17 |
| 269 | PKG | Rp-8-CPT-cGMPS | 101.8 | 91.38 | -10.42 |
| 270 | PKG | KT5823 | 78.59 | 84.43 | 5.84 |
| 271 | PKR | PKR inhibitor | 34.55 | 43.63 | 9.08 |
| 272 | Raf | RAF1 kinase inhibitor I | 97.5 | 101 | 3.5 |
| 273 | Raf | ZM 336372 | 95.74 | 90.24 | -5.5 |
| 274 | ROCK | H-1152 | 68.57 | 80.79 | 12.22 |
| 275 | ROCK | Y-27632 | 90.57 | 92.82 | 2.25 |
| 276 | Hsp90 | radicicol | 30.18 | 48.02 | 17.84 |
| 277 | Src | PP1 analog | 101.33 | 93.45 | -7.88 |
| 278 | Syk | Syk inhibitor | 62.99 | 82.75 | 19.76 |
| 279 | TGF-βRI | SB431542 | 96.65 | 99.51 | 2.86 |
| 280 | TGF-βRI | TGF-b RI kinase inhibitor II | 93.76 | 93.63 | -0.13 |
| 281 | Tpl2 | Tpl2 kinase inhibitor | 93.4 | 88.23 | -5.17 |
| 282 | TrKA | TrkA inhibitor | 86.07 | 85.73 | -0.34 |
| 283 | VEGFR | VEGFR receptor tyrosine kinase inhibitor II | 74.39 | 71.97 | -2.42 |
| 284 | VEGFR | VEGF recptor 2 kinase inhibitor I | 90.78 | 87.74 | -3.04 |
| 285 | VEGFR | SU1498 | 93.22 | 79.94 | -13.28 |
| 286 | Bcr-Abl | nilotinib | 96.86 | 98.37 | 1.51 |
| 287 | Multi-kinases | sorafenib | 15.45 | 20.15 | 4.7 |
| 288 | mTOR | temsirolimus | 88.41 | 84.47 | -3.94 |
| 289 | EGFR/Her2 | lapatinib | 68.01 | 68.35 | 0.34 |
| 290 | Bcr-Abl/Kit | imatinib mesylate | 95.2 | 92.41 | -2.79 |
| 291 | Multi-kinases | sunitinib malate | 24.38 | 40.86 | 16.48 |
| 292 | EGFR | gefitinib | 94.49 | 95.76 | 1.27 |
| 293 | HDAC | vorinostat | 35.69 | 47.75 | 12.06 |
| 294 | EGFR | erlotinib | 93.7 | 96.86 | 3.16 |
| 295 | Proteasome | bortezomib | 24.04 | 40.34 | 16.3 |
| 296 | Bcr-Abl/Src | dasatinib | 42.38 | 64.23 | 21.85 |
| 297 | mTOR | everolimus | 72.72 | 78.57 | 5.85 |
| 298 | Multi-kinases | pazopanib | 32.89 | 49.22 | 16.33 |
| 299 | Rho/SRF | CCG-1423 | 90.14 | 89 | -1.14 |
| 300 | PIM | PIM1/2 Kinase Inhibitor V | 95.34 | 95.54 | 0.2 |
| 301 | PIM | PIM1 Inhibitor II | 110.86 | 109.85 | -1.01 |
| 302 | Hedgehog | AY 9944 | 104.36 | 104.68 | 0.32 |
| 303 | Hedgehog | cyclopamine | 94.36 | 93.47 | -0.89 |
| 304 | Hedgehog | Jervine | 97.14 | 95.25 | -1.89 |
| 305 | STAT3 | WP1066 | 33.25 | 49.03 | 15.78 |
| 306 | STAT3 | 5,15-DPP | 88.79 | 96.07 | 7.28 |
| 307 | Wnt | IWP-2 | 102.69 | 97.25 | -5.44 |
| 308 | Wnt | IWR-1-endo | 93.27 | 96.13 | 2.86 |
| 309 | Wnt | FH535 | 92.25 | 111.9 | 19.65 |
| 310 | Notch | DAPT | 98.69 | 101 | 2.31 |
| 311 | tankyrase-selective PARP | XAV939 | 90.63 | 99.2 | 8.57 |
| 312 | pan-PARP | PJ-34 | 88.49 | 93.12 | 4.63 |
| 313 | PARP-1/2-selective | Olaparib | 61.55 | 81.12 | 19.57 |
| 314 | antipsychotic drug | chlorpromazine hydrochloride | 45.16 | 65.12 | 19.96 |
| 315 | depression treatment | desipramine hydrochloride | 92.68 | 92.85 | 0.17 |
| 316 | golgi inhibitor | brefeldin A | 33.68 | 50.54 | 16.86 |
| 317 | stress inducer | anisomycin | 35.27 | 49.49 | 14.22 |
| 318 | thalidomide family | thalidomide | 102.84 | 105.98 | 3.14 |
| 319 | thalidomide family | lenalidomide | 103.66 | 102.69 | -0.97 |
| 320 | retinoids | tretinoin | 96.56 | 96.11 | -0.45 |
| 321 | retinoids | tamibarotene | 96.98 | 100.98 | 4 |
| 322 | DNA alkylation | temozolomide | 100.07 | 101.91 | 1.84 |
| 323 | EML4-ALK | crizotinib | 13.15 | 20.42 | 7.27 |
| 324 | mTOR | Torkinib | 106.02 | 99.16 | -6.86 |
| 325 | lipase | orlistat | 58.79 | 72.96 | 14.17 |
| 326 | AR | MDV3100 | 101.82 | 99.76 | -2.06 |
| 327 | caspase activator | PAC-1 | 39 | 59.7 | 20.7 |
| 328 | blc-2 | ABT-737 | 45.67 | 42.1 | -3.57 |
| 329 | G9a | UNC0638 | 20.22 | 27.18 | 6.96 |
| 330 | G9a | BIX01294 | 30.08 | 37.32 | 7.24 |
| 331 | LSD1 | S2101 (LSD1 inhibitor II) | 97.78 | 96.76 | -1.02 |
| 332 | PRMT1 | AMI-1 | 98.38 | 101.33 | 2.95 |
| 333 | p300 | C646 | 102.23 | 92.12 | -10.11 |
| 334 | SIRT1 | SIRT1 inhibitor III | 79.64 | 103 | 23.36 |
| 335 | SIRT1/2 | Tenovin-6 | 27.2 | 33.55 | 6.35 |
| 336 | HDAC8 | PCI-34051 | 79.4 | 92.85 | 13.45 |
| 337 | BRD4 bromodomain | (+)-JQ1 | 41.9 | 50.21 | 8.31 |
| 338 | Telomerase | TMPyP4 | 96.09 | 97.79 | 1.7 |
| 339 | PARP | BSI-201 (Iniparib) | 110.67 | 105.86 | -4.81 |
| 340 | PARP | ABT-888 (Veliparib) | 93.94 | 97.03 | 3.09 |
| 341 | PARP | AG014699 (Rucaparib) | 73.51 | 79.61 | 6.1 |
| 342 | PARP | MK-4827 (Niraparib) | 65.98 | 83.64 | 17.66 |
| 343 | Aurora | ENMD-2076 | 106.38 | 104.84 | -1.54 |
| 344 | Aurora | MLN8237 | 98.21 | 95.33 | -2.88 |
| 345 | Survivin | YM155 | 12.37 | 14.94 | 2.57 |
| 346 | PDK1 | OSU-03012 | 88.14 | 103.25 | 15.11 |
| 347 | IGF-IR | OSI-906 | 68.77 | 75.67 | 6.9 |
| 348 | c-Met | PF-04217903 | 91.84 | 97.25 | 5.41 |
| 349 | DNMT | Decitabine | 98.16 | 97.27 | -0.89 |
| 350 | Multi-kinases | Vandetanib | 44.5 | 64.54 | 20.04 |
| 351 | Multi-kinases | Axitinib | 62.33 | 68.6 | 6.27 |
|  |  |  |  |  |  |
| 352 | BRAF | Vemurafenib | 67.58 | 96.07 | 28.49 |
| 353 | JAK | Ruxolitinib | 65.06 | 84.34 | 19.28 |
| 354 | Hedgehog | Vismodegib | 101.13 | 102.36 | 1.23 |
| 355 | GLI1 | Gant61 | 99.4 | 94.17 | -5.23 |
| 356 | FGFR | PD173074 | 40.32 | 54.47 | 14.15 |
| 357 | ALK | A83-01 | 91.87 | 94.03 | 2.16 |
| 358 | GSK-3 | BIO | 75.72 | 88.98 | 13.26 |
| 359 | GSK-3 | TWS119 | 34.86 | 55.03 | 20.17 |
| 360 | GSK-3 | CT99021 | 86.27 | 97.87 | 11.6 |
| 361 | TGFb-R | LY2157299 | 95.26 | 99.11 | 3.85 |
| 362 | TGFb-R | SD208 | 93.07 | 100.34 | 7.27 |
| 363 | ALK | LDN193189 | 13.36 | 16.9 | 3.54 |
| 364 | ROCK | Thiazovivin | 87.72 | 88.23 | 0.51 |
