# Supplementary material for: CAMK2D: a novel molecular target for BAP1-deficient malignant mesothelioma
Source: Cell Death Discov. 2023 Jul 21;9:257. doi: 10.1038/s41420-023-01552-5 (PMC10362017; doi:10.1038/s41420-023-01552-5)
Supplement: Supplementary file 5 — Table S4. Primer sets used in this study [file 41420_2023_1552_MOESM5_ESM.docx]

Table S4. Primer sets used in this study

| Symbol Forward primer (5′→3′) Reverse primer (5′→3′) |
| --- |

*CAMK2D* CCTACTGGACAAGAATATGC TTAGGGTGCTTCAAAAGACGG

*MFAP4* CGTCTTCTGTGACATGACCA ACTCGCAGCTCATACTTCTG

*NPTX1* GAGGAGAGGGTCAAGATCGA AGCTGGAACTTGTCTCCAGG

*HMGA2* GCAGCAAAAACAAGAGTCCC GGCTTCTTCTGAACAACTTG

*HOXA5* GATGCGCAAGCTGCACATAA TAACGGTTGAAGTGGAACTC

*PRKCZ* GGATGAAGGCCTCATCATTC AAGCGCTTGGCTTGGAAGAG

*CD200* CTGTCTACCTACAGCCTGG GTCACAATGAGGGCTTCCTG

*CD40* GTCGGCTTCTTCTCCAATGT ACATCAGTCTTGTTTGTGCC

*BAP1* GCTCGTGGAAGATTTCGGTGT TCATCAATCACGGACGTATCATC

*GAPDH* GAGTCAACGGATTTGGTCGT GACAAGCTTCCCGTTCTCAG

|  |
| --- |
