# Supplementary material for: CAMK2D: a novel molecular target for BAP1-deficient malignant mesothelioma
Source: Cell Death Discov. 2023 Jul 21;9:257. doi: 10.1038/s41420-023-01552-5 (PMC10362017; doi:10.1038/s41420-023-01552-5)
Supplement: Supplementary file 6 — Table S5. Antibodies used in this study [file 41420_2023_1552_MOESM6_ESM.docx]

Table S5. Antibodies used in this study

| Molecule | Cat number | Company | Species | Dilution |
| --- | --- | --- | --- | --- |
| BAP1 | #13271 | CST | Rb | ×2000 |
| EZH2 | 21800-1-AP | Proteintech | Rb | ×2000 |
| H3K27me3 | # 9733S | CST | Rb | ×1000 |
| Histone H3 | # 4499 | CST | Rb | ×1000 |
| CAMK2D | AP7209a | Abcepta | Rb | ×1000 |
| CAMK2D  Calmodulin | Sc-100362  A4885 | Santa Cruz  ABclonal | Ms  Rb | ×200  ×2000 |
| Phospho-stat3 | #9145 | CST | Rb | ×2000 |
| Total-stat3 | #12640 | CST | Rb | ×3000 |
| CDK2 | #18048 | CST | Rb | ×3000 |
| C-PAPR  Cleaved caspase 3 | #9548  #9661 | CST  CST | Rb  Rb | ×2000  ×2000 |
| GAPDH | #2118 | CST | Rb | ×2000 |
| Anti-rabbit IgG-HRP | #7074 | CST | Goat | ×4000 |

CST, Cell Signaling Technology; Rb, rabbit; Ms, mouse
