# Supplementary material for: CAMK2D: a novel molecular target for BAP1-deficient malignant mesothelioma
Source: Cell Death Discov. 2023 Jul 21;9:257. doi: 10.1038/s41420-023-01552-5 (PMC10362017; doi:10.1038/s41420-023-01552-5)
Supplement: Supplementary file 7 — Table S6. Blood chemistry in vehicle control mice and KN-93-treated mice [file 41420_2023_1552_MOESM7_ESM.docx]

Table S6. Blood chemistry in vehicle control mice and KN-93-treated mice

| Test | Unit | Control | KN-93- treated  (15mg/kg, 6 times injection) |
| --- | --- | --- | --- |
|  |  | (Mean ± SD) | (Mean ± SD) |
| TP | g/dL | 4.20 ± 0.04 | 3.90± 0.07 |
| ALB | g/dL | 2.84 ± 0.02 | 2.74 ± 0.05 |
| BUN | mg/dL | 21.62 ± 0.54 | 20.98 ± 1.10 |
| CRE | mg/dL | 0.12 ± 0.01 | 0.15 ± 0.01 |
| Na | mEq/L | 149.80 ± 0.80 | 150.40 ± 0.73 |
| K | mEq/L | 5.30 ± 0.18 | 5.08 ± 0.20 |
| Cl | mEq/L | 110.40 ± 0.40 | 112.80 ± 0.52 |
| Ca | mg/dL | 8.28 ± 0.31 | 8.10 ± 0.14 |
| IP | mg/dL | 12.52 ± 0.97 | 14.80 ± 0.67 |
| AST | IU/L | 48.60 ± 2.66 | 53.20 ± 1.66 |
| ALT | IU/L | 26.60 ± 2.25 | 30.60 ± 1.40 |
| LDH | IU/L | 148.60 ± 13.04 | 158.80 ± 16.73 |
| AMY | IU/L | 2511.20 ± 122.93 | 2113.2 ± 78.11 |
| γ-GT | IU/L | 3＞ | 3＞ |
| T-CHO | mg/dL | 76.40 ± 2.89 | 68.60 ± 1.34 |
| TG | mg/dL | 93.40 ± 11.98 | 105.40 ± 11.09 |
| HDL-C | mg/dL | 39.00 ± 1.00 | 34.80 ± 0.66 |
| T-BIL | mg/dL | 0.06 ± 0.004 | 0.05 ± 0.005 |
| GLU | mg/dL | 128.60 ± 18.74 | 157.60 ± 21.19 |

**Abbreviations:** TP, total protein; ALB, albumin; BUN, blood urea nitrogen; CRE, creatinine; Na, sodium; K, potassium; Cl, chloride; Ca, calcium; IP, inorganic phosphorus; AST, aspartate aminotransferase; ALT, alanine aminotransferase; LDH, lactate dehydrogenase; AMY, amylase; γ-GT, gamma-glutamyl transferase; T-CHO, total cholesterol; TG, triglycerides; HDL-C, high-density lipoprotein cholesterol; T-BIL, total bilirubin; GLU, glucose (blood sugar); bw, body weight
