# Supplementary figures and images for: CAMK2D: a novel molecular target for BAP1-deficient malignant mesothelioma
Source: Cell Death Discov. 2023 Jul 21;9:257. doi: 10.1038/s41420-023-01552-5 (PMC10362017; doi:10.1038/s41420-023-01552-5)

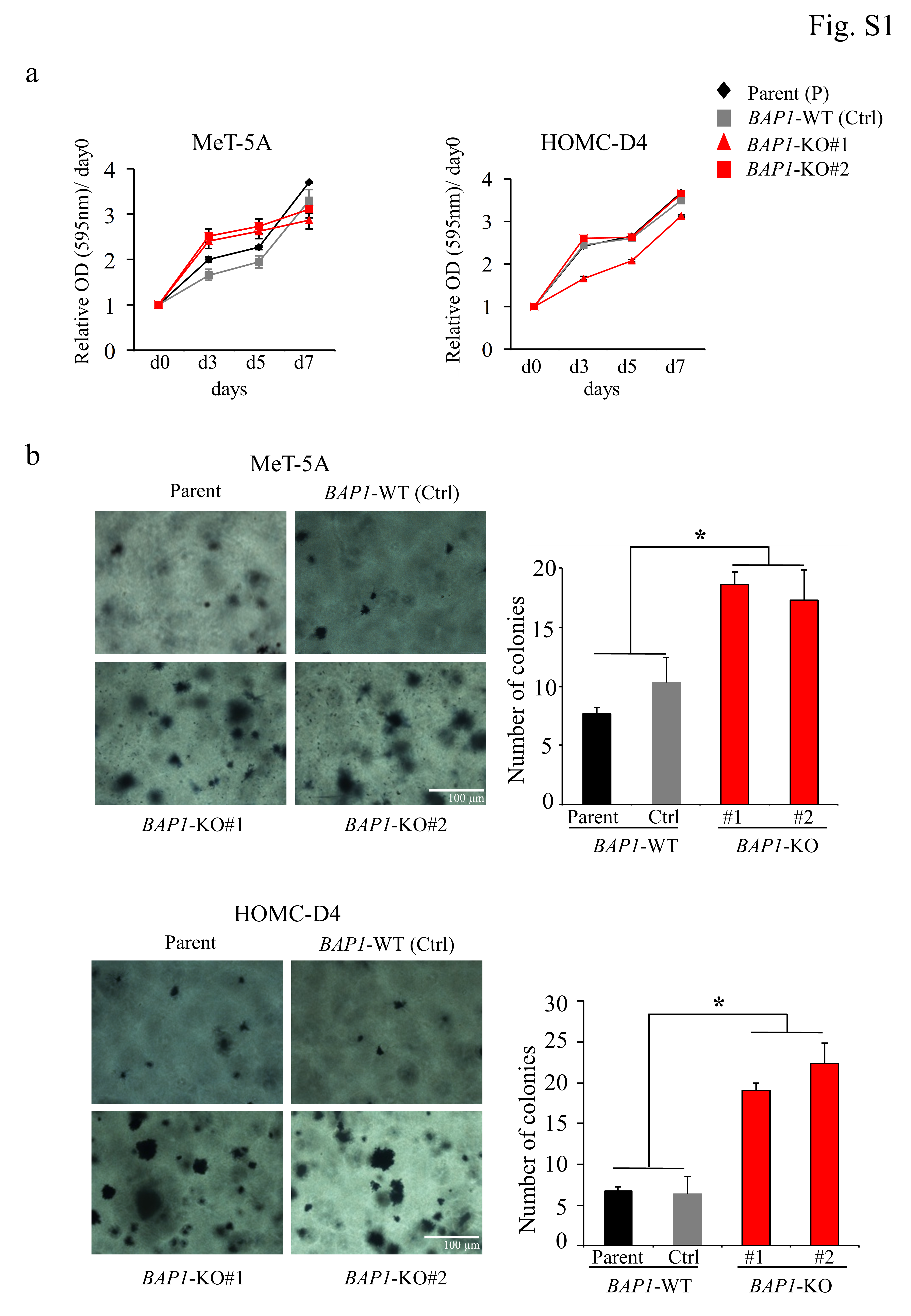

Supplement: Supplementary file 8 — Fig. S1.Effect of BAP1 loss on the proliferation and colony formation in MeT-5A and HOMC-D4 cells [file 41420_2023_1552_MOESM8_ESM.tif]

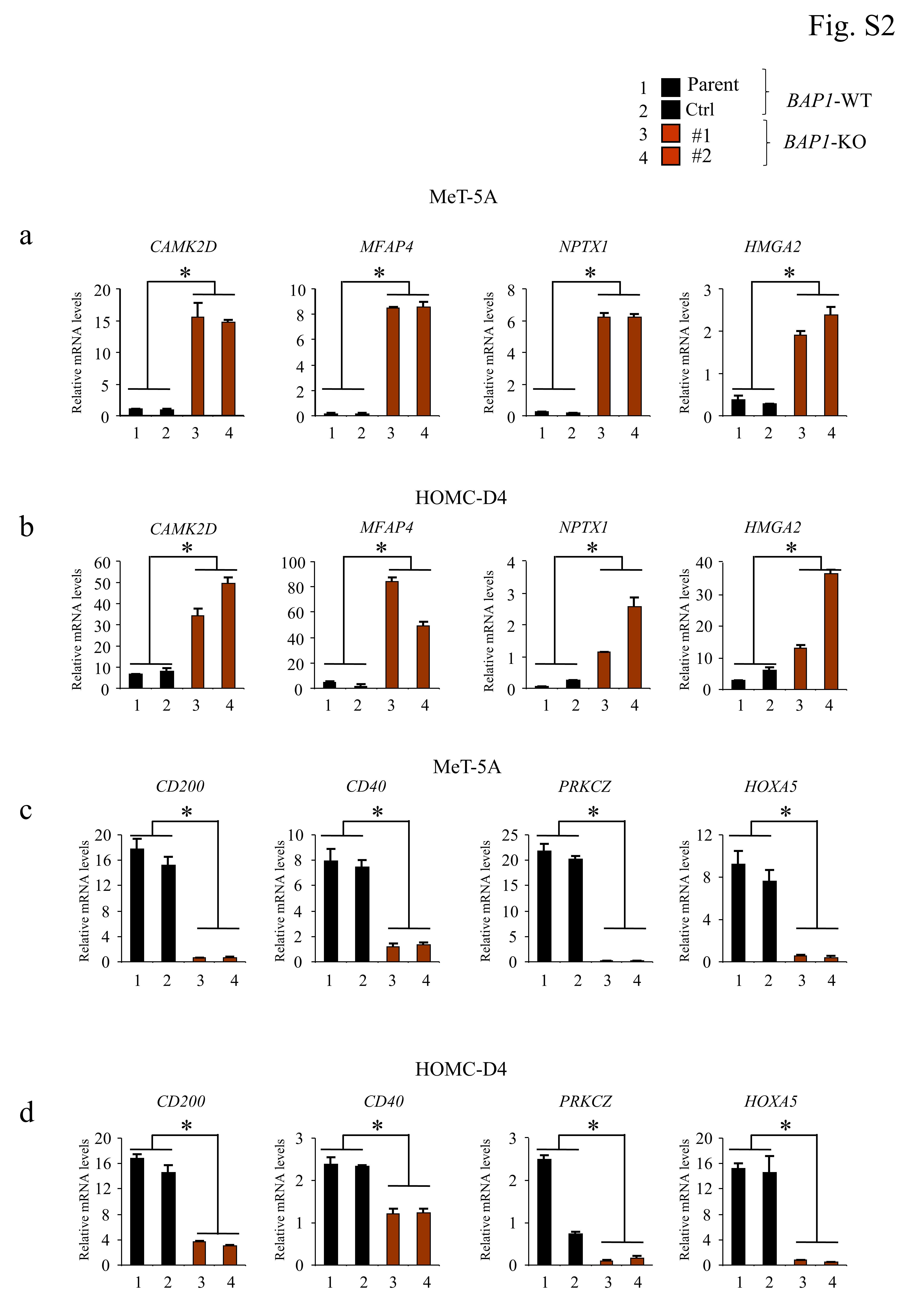

Supplement: Supplementary file 9 — Fig. S2. Effect of BAP1 loss on the gene expression in MeT-5A and HOMC-D4 cells [file 41420_2023_1552_MOESM9_ESM.tif]

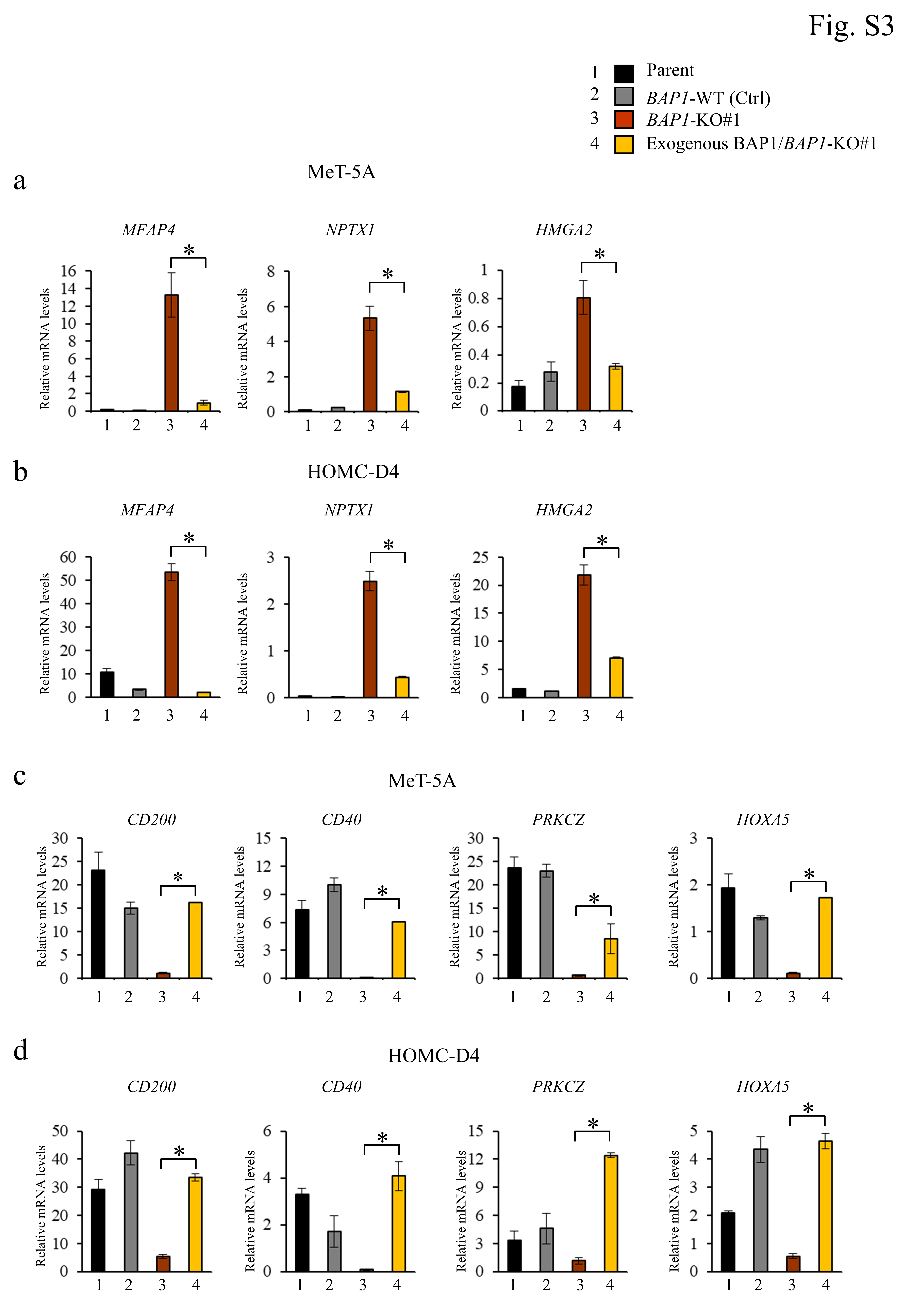

Supplement: Supplementary file 10 — Fig. S3. Effect of exogenous BAP1 on the gene expression [file 41420_2023_1552_MOESM10_ESM.tif]

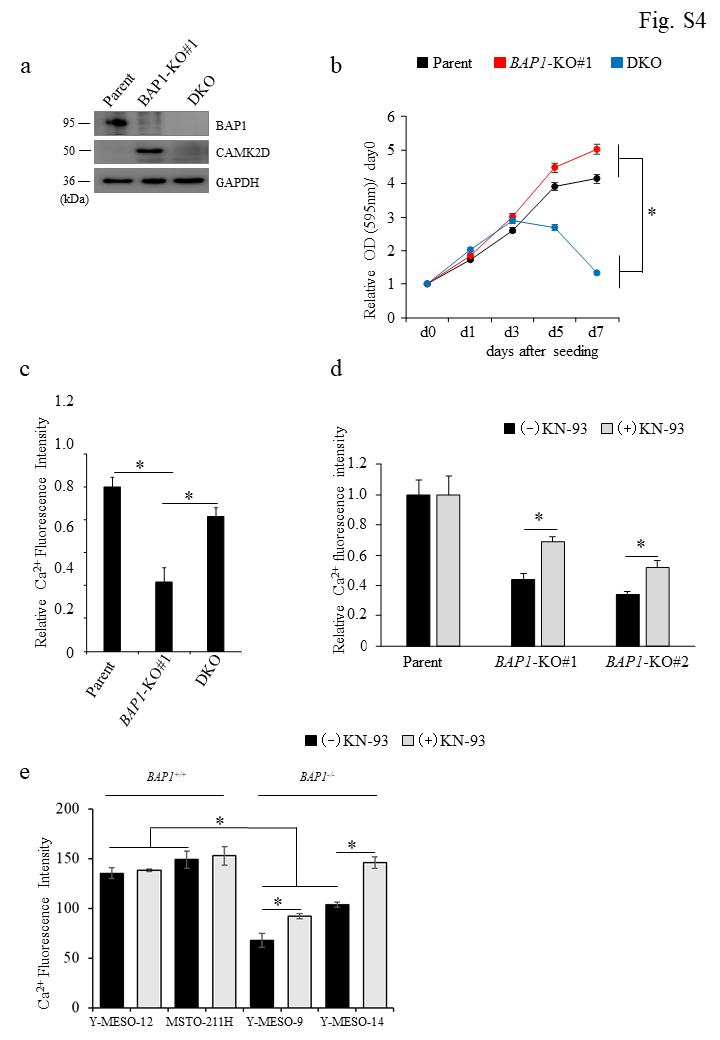

Supplement: Supplementary file 11 — Fig. S4. Effect of BAP1 loss and KN-93 treatment on intracellular Ca2+ levels [file 41420_2023_1552_MOESM11_ESM.tif]

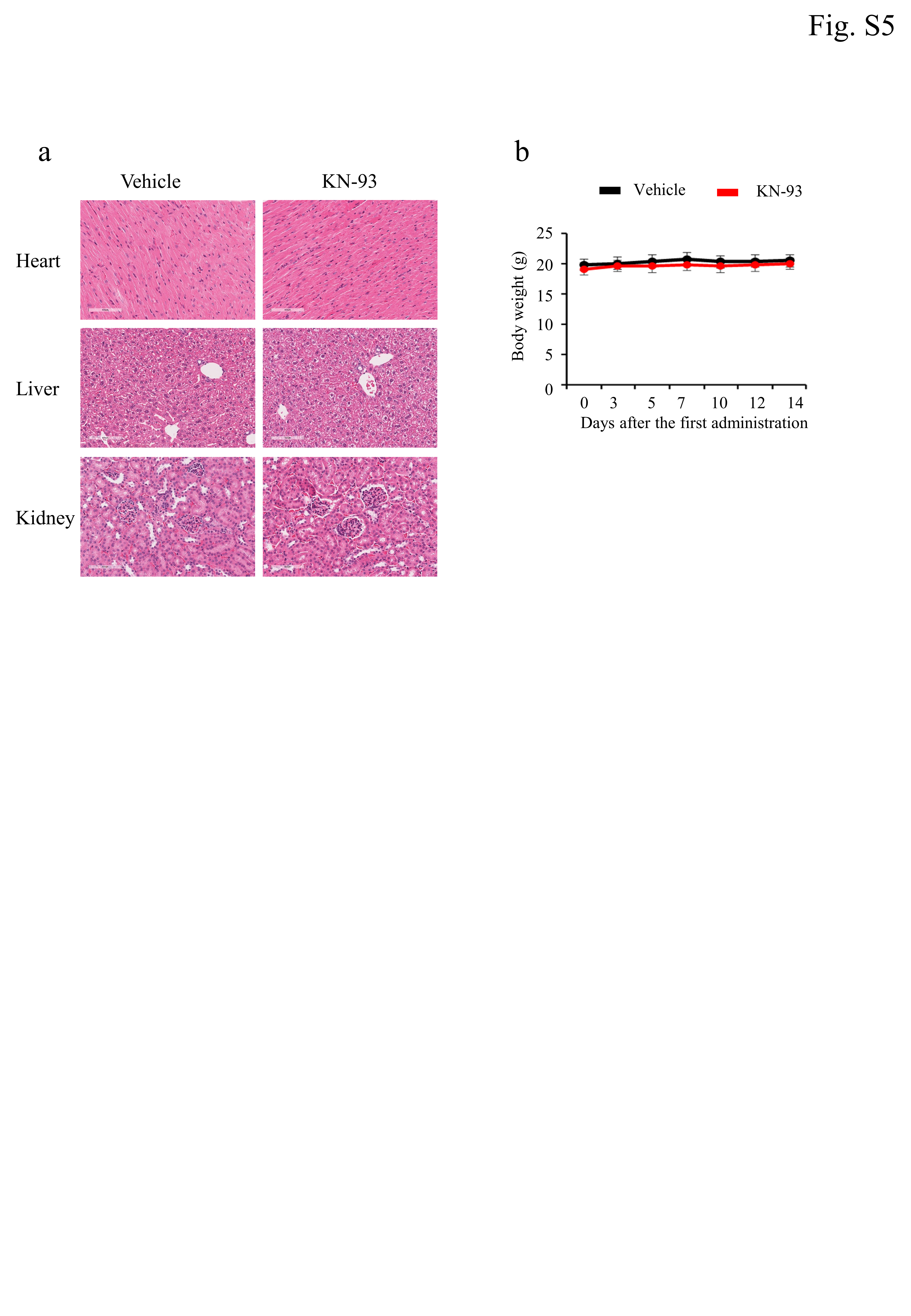

Supplement: Supplementary file 12 — Fig. S5. In vivo effects of KN-93 on BALB/cCrSlc mice [file 41420_2023_1552_MOESM12_ESM.tif]

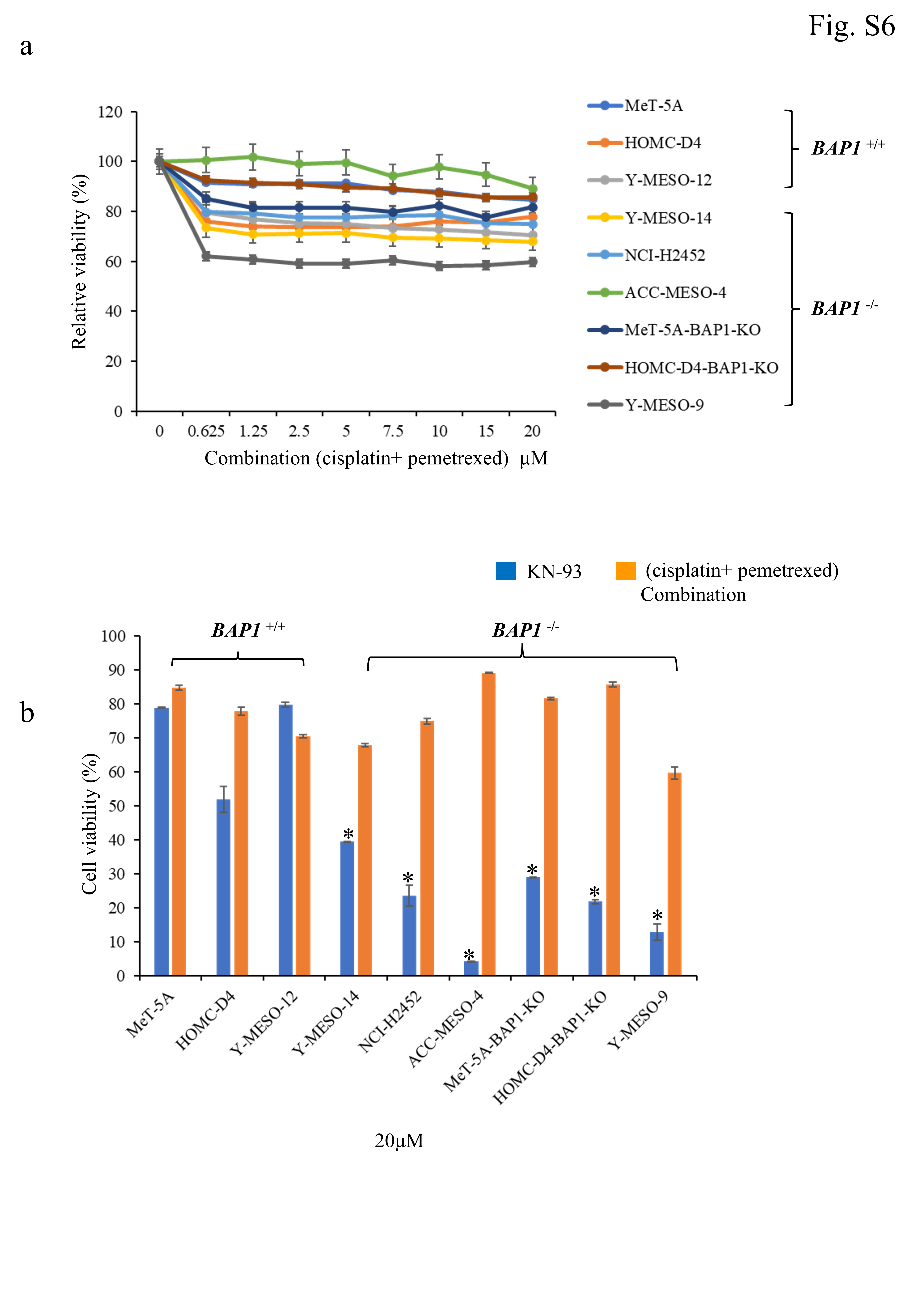

Supplement: Supplementary file 13 — Fig. S6. Effect of cisplatin and pemetrexed combination treatment on cell viability [file 41420_2023_1552_MOESM13_ESM.tif]

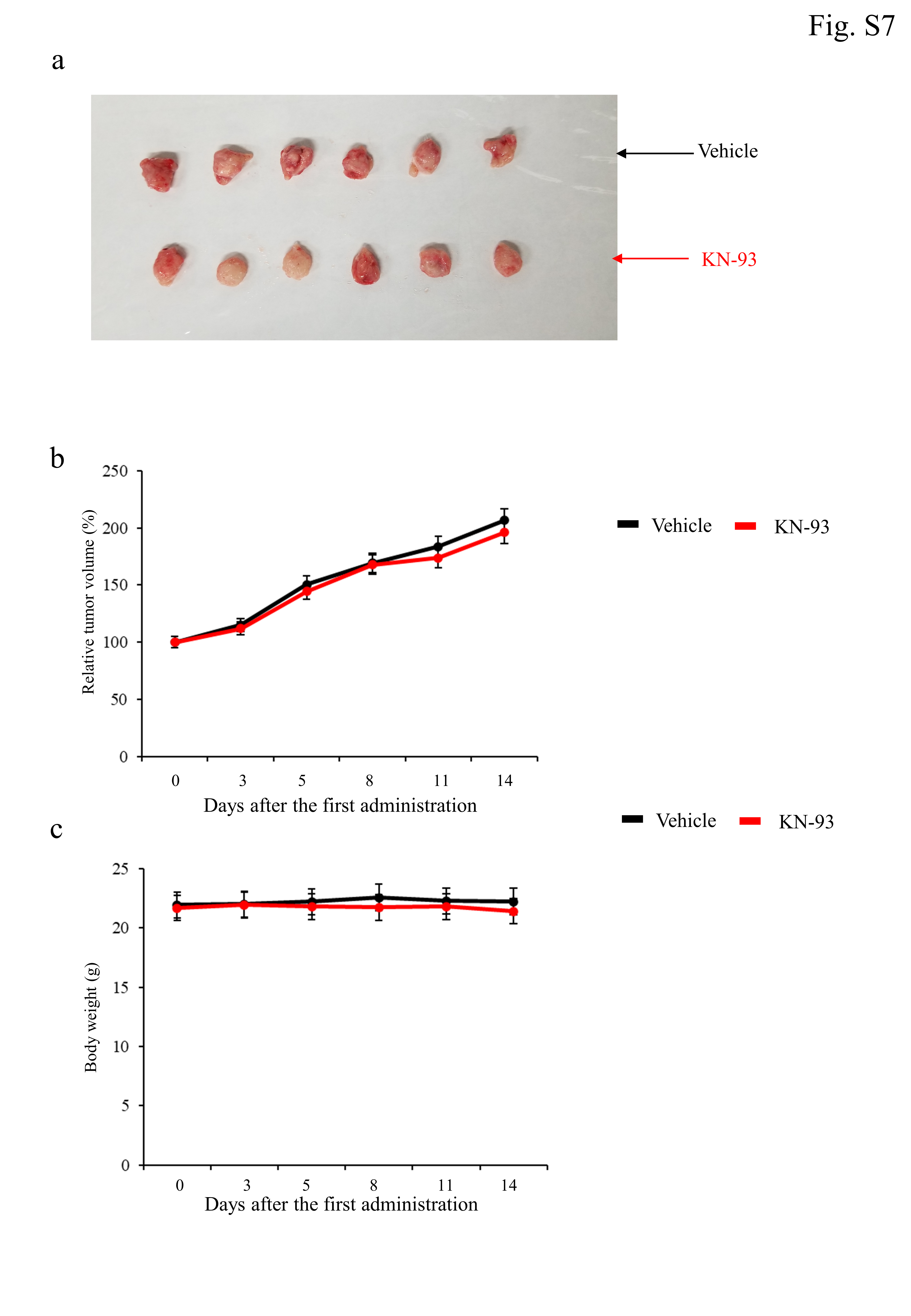

Supplement: Supplementary file 14 — Fig. S7. Effect of KN-93 on the tumor growth of MSTO-211H cells in vivo [file 41420_2023_1552_MOESM14_ESM.tif]

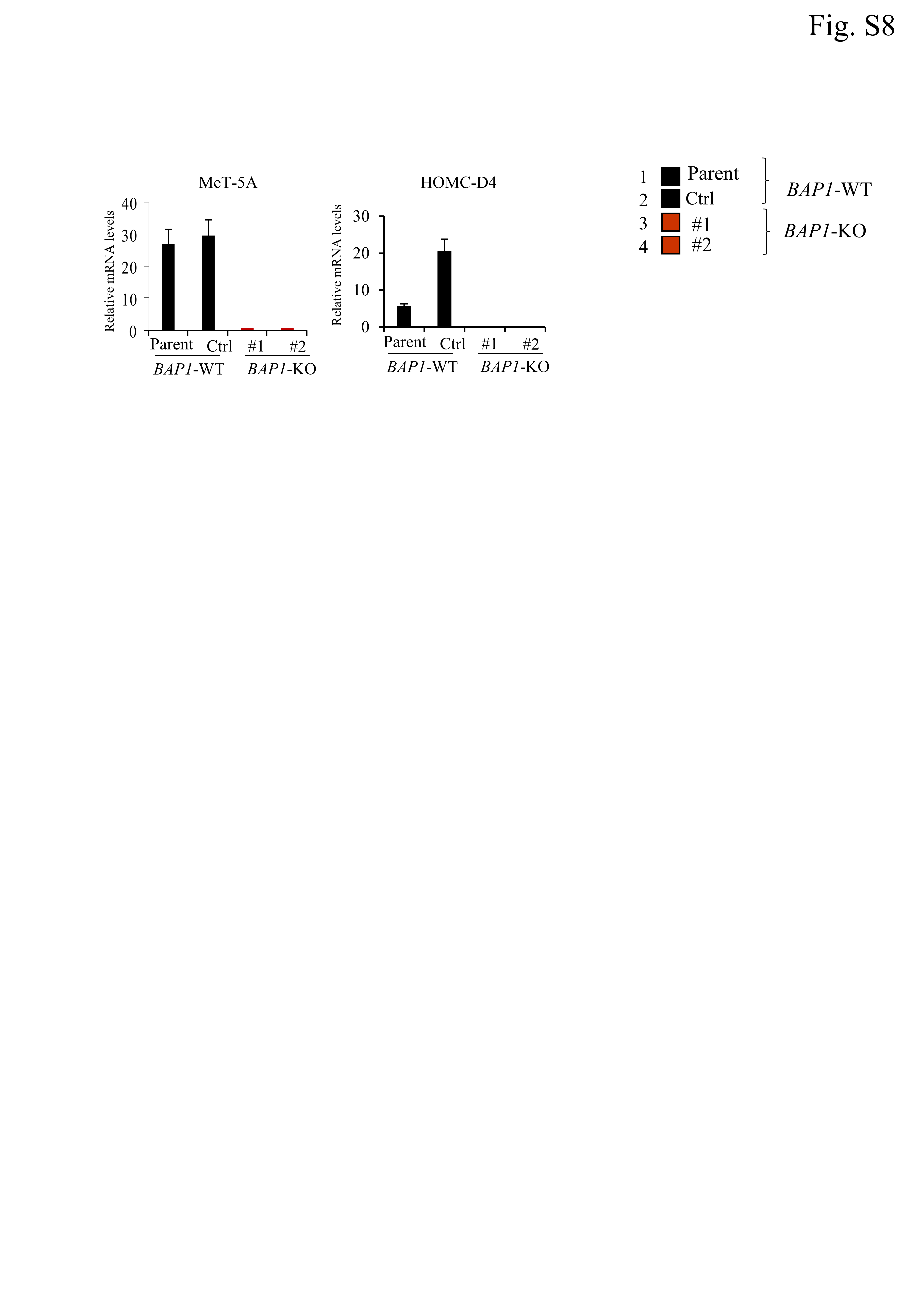

Supplement: Supplementary file 15 — Fig. S8. mRNA expression of BAP1 in MeT-5A and HOMC-D4 cells [file 41420_2023_1552_MOESM15_ESM.tif]
