# Supplementary material for: CAMK2D: a novel molecular target for BAP1-deficient malignant mesothelioma
Source: Cell Death Discov. 2023 Jul 21;9:257. doi: 10.1038/s41420-023-01552-5 (PMC10362017; doi:10.1038/s41420-023-01552-5)

Full and uncropped western blots image of Fig. 1b

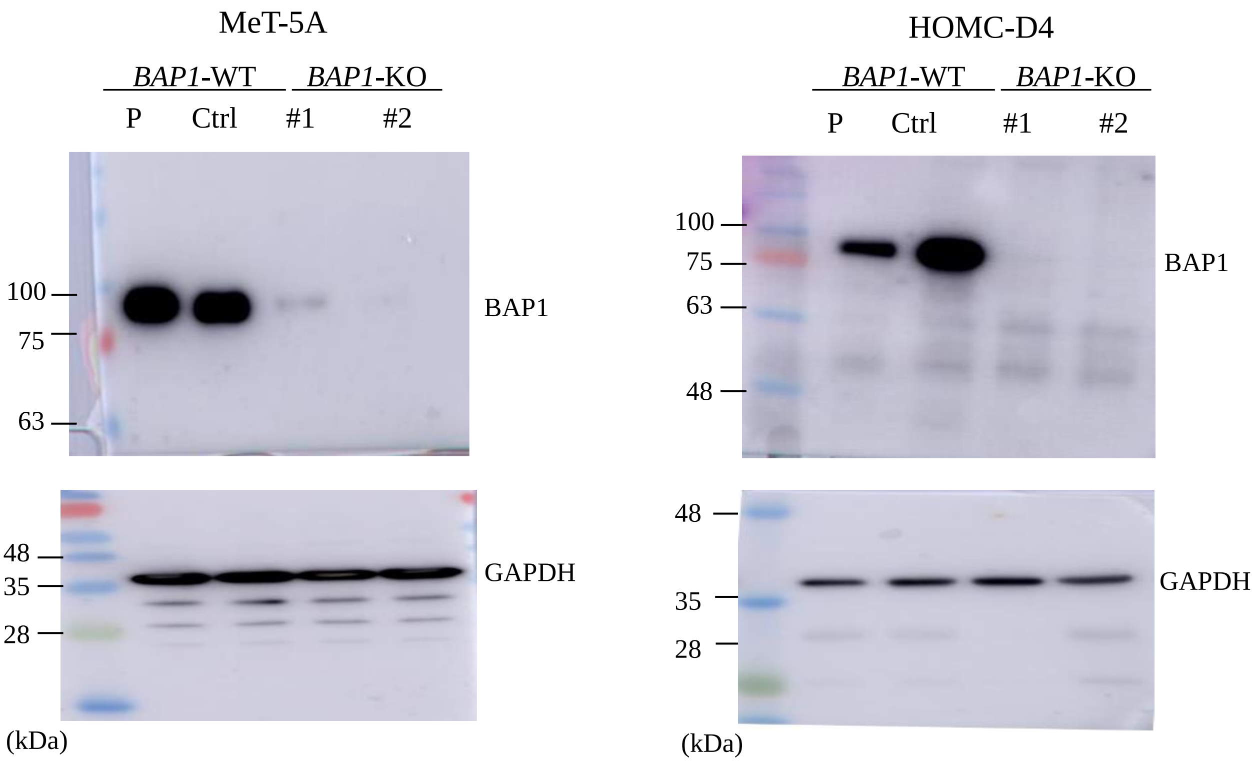

Full and uncropped western blots image of Fig. 2b

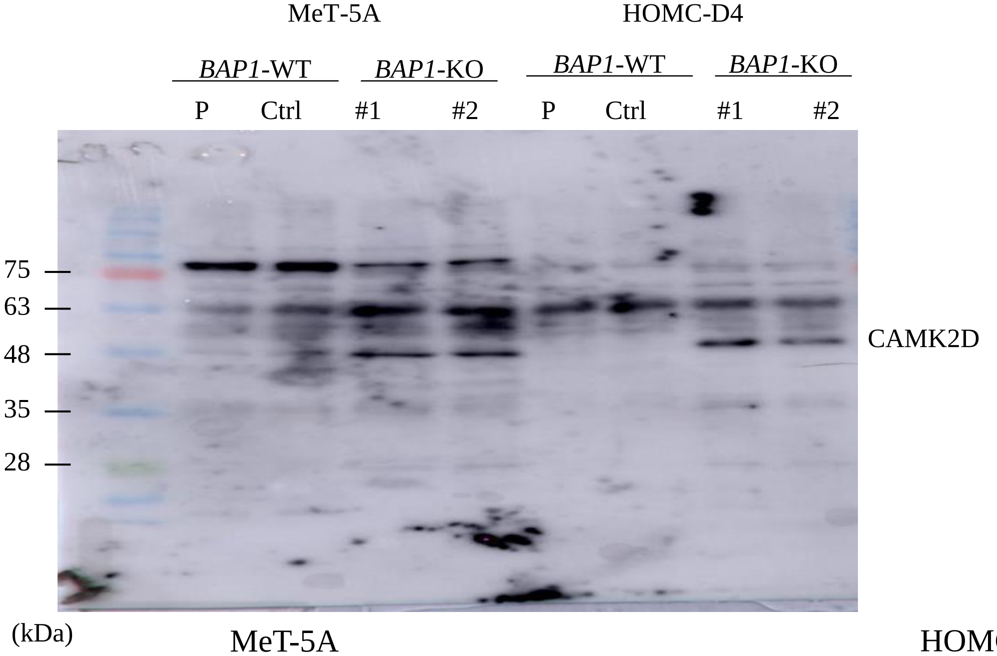

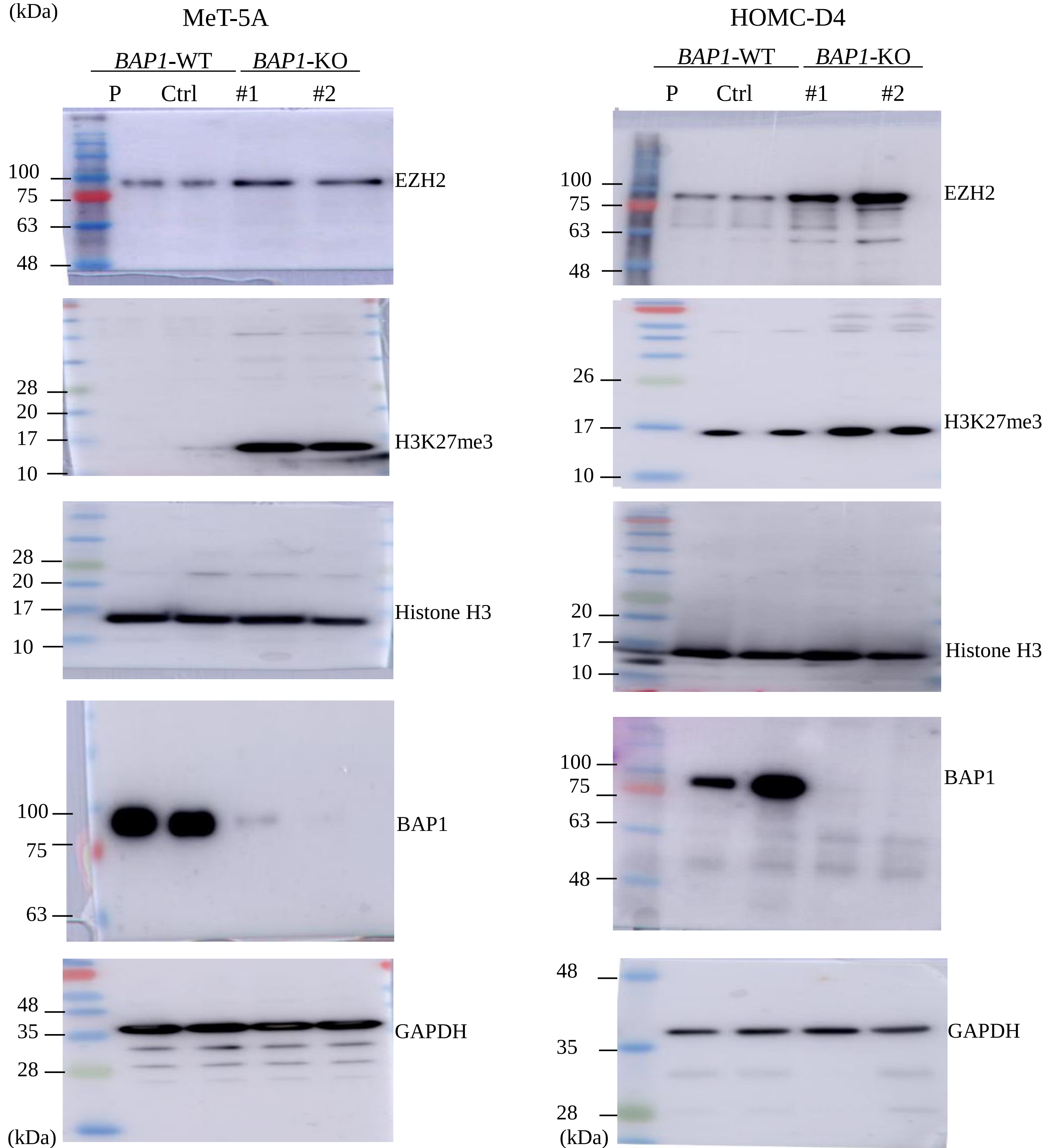

Full and uncropped western blots image of Fig. 2 c

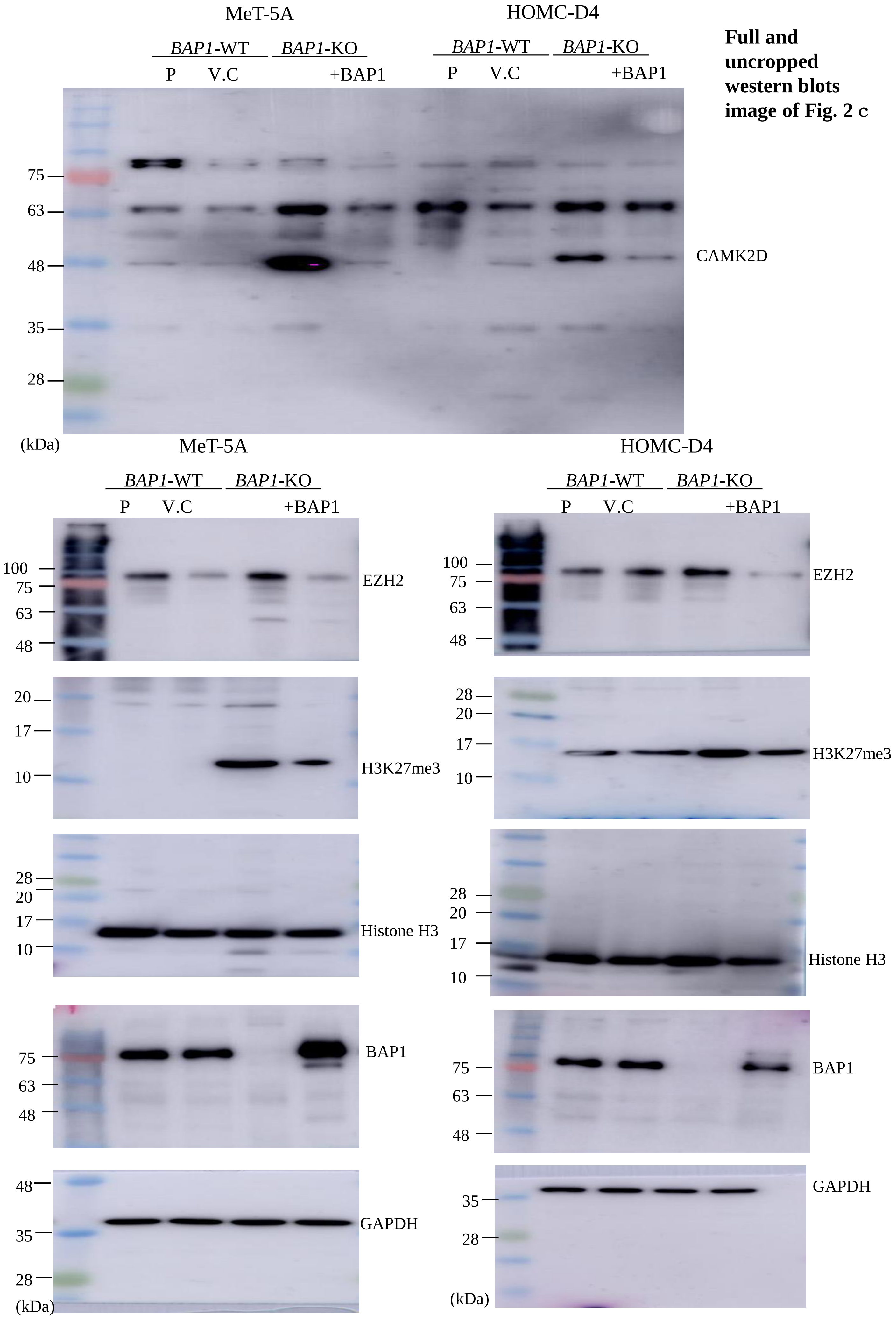

Full and uncropped western blots image of Fig. 4b

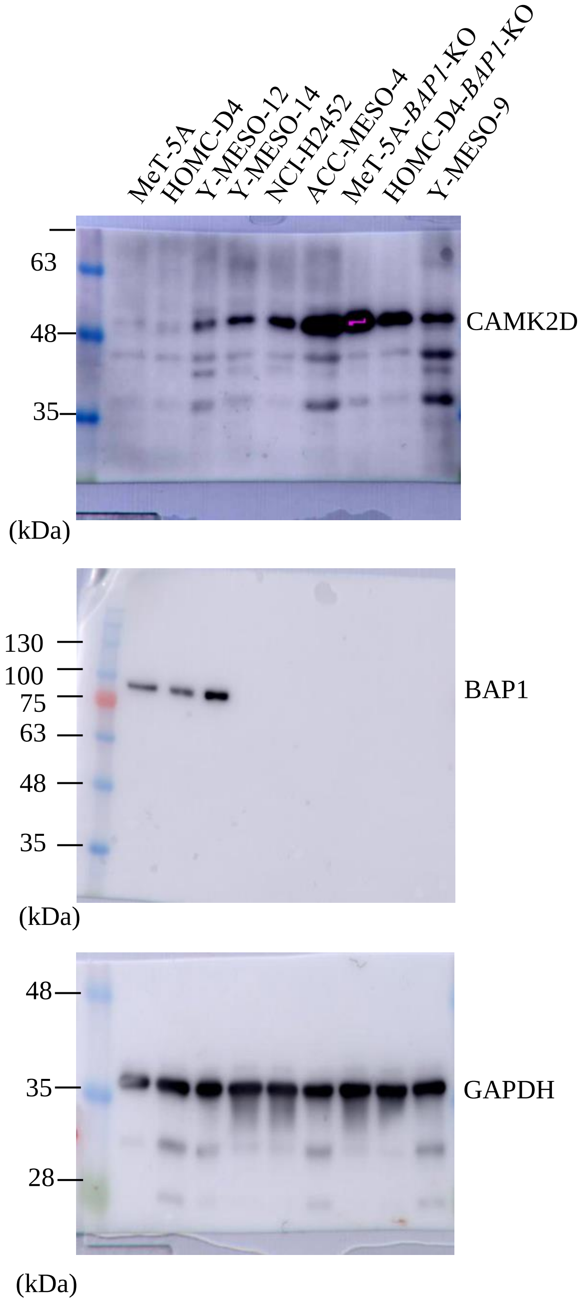

Full and uncropped western blots image of Fig. 5b

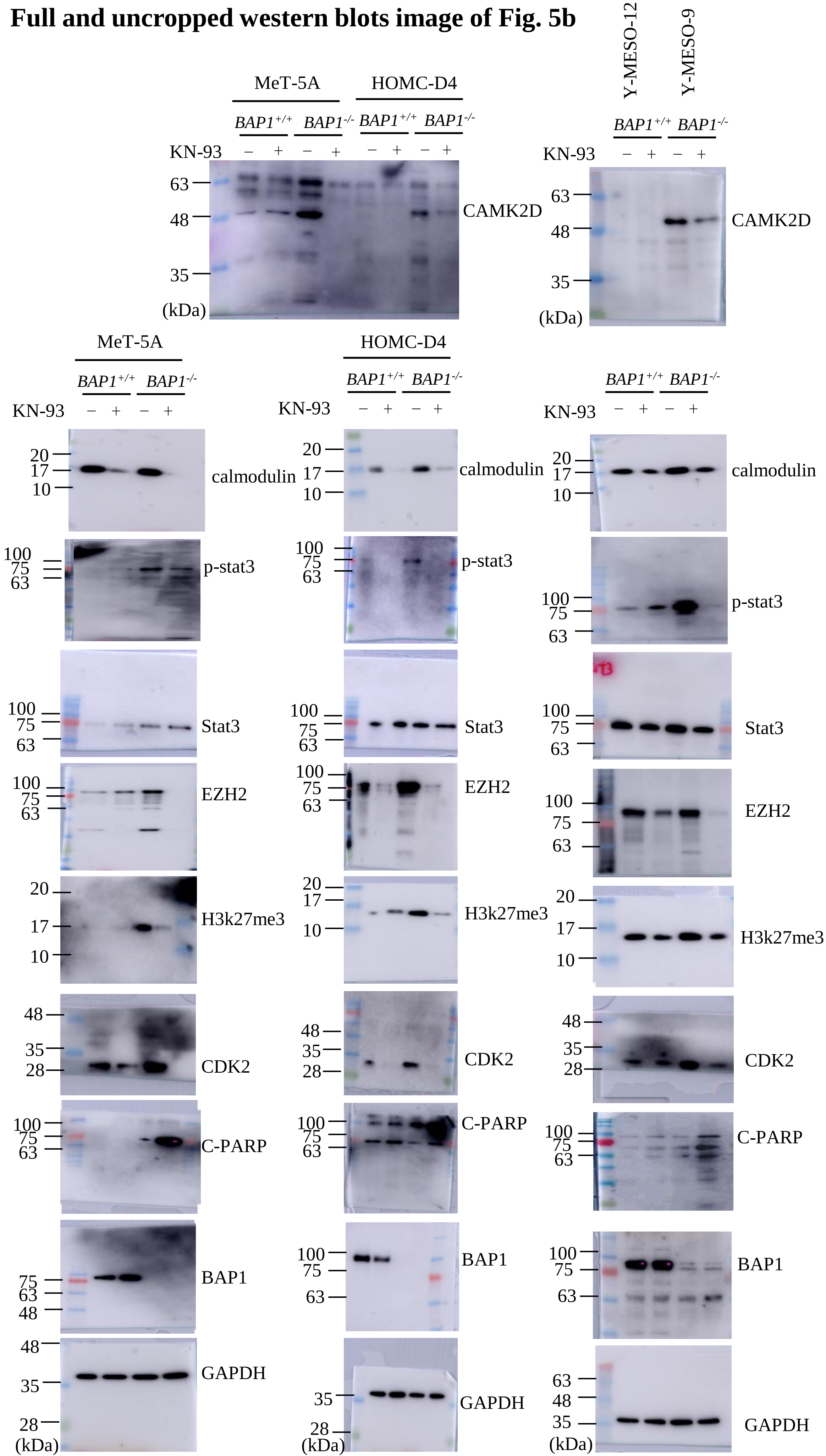

Full and uncropped western blots image of Fig. 6d

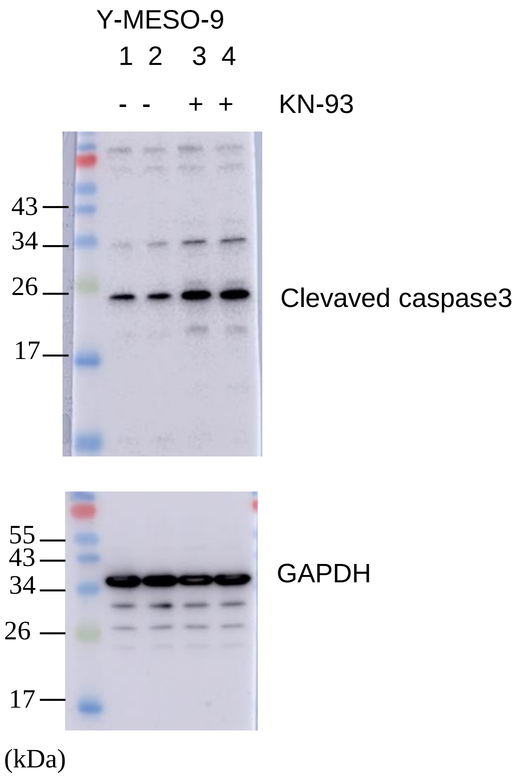

Full and uncropped western blots image of Fig. S4

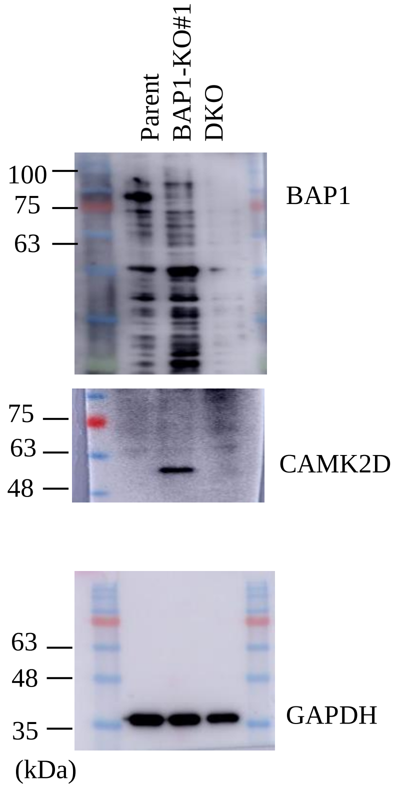

Supplement: Supplementary file 16 — Full and uncropped western blots images [file 41420_2023_1552_MOESM16_ESM.pdf]
